# Prepartum working conditions predict mental health symptoms 14 months postpartum in first-time mothers and their partners – results of the prospective cohort study “DREAM”

**DOI:** 10.1186/s12889-025-21886-2

**Published:** 2025-03-05

**Authors:** Lydia Rihm, Jasmin Waibel, Marlene Karl, Judith T. Mack, Victoria Weise, Susan Garthus-Niegel

**Affiliations:** 1 Institute for Systems Medicine, Faculty of Medicine, MSH Medical School Hamburg – University of Applied Sciences and Medical University, Hamburg, Germany; 2 Institute and Policlinic of Occupational and Social Medicine, Faculty of Medicine, TUD Dresden University of Technology, Dresden, Germany; 3 Department of Child and Adolescent Psychiatry, TUD Dresden University of Technology, Dresden, Germany; 4 Department of Childhood and Families, Norwegian Institute of Public Health, Oslo, Norway

**Keywords:** DREAM study, Peripartum/perinatal mental health, Fathers’ mental health, Postpartum depression, Working conditions, Precarious employment, Abusive supervision, Job insecurity, Job demand, Work stress

## Abstract

**Background:**

During the vulnerable transition to parenthood, (expectant) parents may be particularly susceptible to the negative effects of adverse working conditions. However, research on the influence of work-related factors on peripartum mental health issues is scarce. This study aims to enhance our understanding of work-related risk factors for the adjustment of parents in the transition to parenthood by investigating the role of prepartum precarious employment, abusive supervision, job insecurity, and job demand on postpartum mental health symptoms in first-time mothers and their partners.

**Methods:**

In the prospective-longitudinal cohort study “DREAM”, *N* = 1,259 mothers and *N* = 811 male and female partners were asked about their working conditions during pregnancy and their mental health 14 months postpartum. We conducted several hierarchical multiple regression analyses with prepartum precarious employment, abusive supervision, job insecurity, and job demand (the latter three in joint regression analyses) as predictors of postpartum symptoms of depression, somatization, obsessive-compulsiveness, anxiety, and anger/hostility. In Model 1 we controlled for sociodemographic variables, and in Model 2 we also controlled for pre-existing symptoms of the respective mental health variable during pregnancy. Separate analyses were calculated for mothers and partners, and each mental health outcome.

**Results:**

Multiple regression analyses revealed that prepartum precarious employment and abusive supervision predicted mothers’ and partners’ mental health symptoms 14 months postpartum even after controlling for pre-existing symptoms. Prepartum job insecurity and job demand also predicted mental health symptoms 14 months postpartum but were no longer significant predictors in most models after controlling for pre-existing mental health symptoms. There were only minor differences regarding the considered mental health outcomes and between mothers’ and partners’ results.

**Conclusions:**

Our study demonstrates that adverse working conditions are important risk factors for the adjustment of parents in the transition to parenthood, requiring more attention from research and practice. Precarious employment and abusive supervision appear to be particularly important factors affecting new parents’ mental health. Future research should investigate the mechanisms behind these variables, including comparisons between mothers and their partners, and the role of stress-related biomarkers. Additionally, developing screening methods for clinical use to facilitate targeted preventive interventions is essential.

**Supplementary Information:**

The online version contains supplementary material available at 10.1186/s12889-025-21886-2.

## Background

Labor work constitutes a major aspect of adult life, exerting a profound and intricate impact on well-being [1, 2]. While employment has been found to be overall beneficial for workers’ mental health [3], poor working conditions, including low levels of control, high job demand, job insecurity, and unfair pay, can have significant adverse effects on mental health [4–7] – potentially even more detrimental than unemployment [4, 8]. Poor mental health in the workplace is not only a major burden for affected individuals and their families, but also for society as a whole, as mental health problems are a leading cause of loss of productivity and reduced work ability [9–12] as well as high global economic costs [13]. Consequently, identifying and mitigating working conditions that impede employees’ mental health represents a public mental health concern and is increasingly a focus of research [5, 6].

A significant proportion of the global workforce consists of parents: In 2023, 34% of prime-aged adults (aged 25 to 54) had at least one child *under the age of six* [14], and the vast majority of adults (around 80–90% across OECD countries) will eventually transition to parenthood [15]. Furthermore, dual-income families are becoming more common around the world [16], which is increasing the number of working parents. However, as the transition to parenthood is considered a vulnerable period with many challenges [17], expectant parents may be particularly susceptible to developing mental health issues in adverse working environments. Although there is a growing body of research on mental health in the workplace on the one hand and mental health during the transition to parenthood on the other, the two research areas have rarely been combined [18–20]. In order to inform work policies that maintain the mental health and working ability of (expectant) parents [21], it is of central importance to clarify the role of working conditions on the mental health of parents in the transition to parenthood.

### Mental health in the transition to parenthood

Despite the joy of welcoming a new baby, the transition to parenthood is a phase with numerous physiological, psychological, economic, and social challenges for new parents [17, 22]. Alongside the (physical) strains of pregnancy and childbirth mostly mothers carry [22–24], both mothers and their partners must fundamentally restructure their lives to adapt to their new roles as parents and adjust to the infants’ needs [25–27]. Sleep disturbances, relationship strain, and heightened emotional concerns compound the experience [25–28]. Whereas many mothers and partners experience personal growth in their new role as parents (e.g., [29]), the added stressors and challenges can surpass available resources and coping mechanisms, hindering successful adjustment [17]. Consequently, the time after childbirth is often accompanied by the onset of mental health problems [17]. Of these, postpartum depression (global estimate of prevalence among mothers: 17.2–27.6%, [30, 31], and among fathers: 5.6–8.8%, [32, 33]) and postpartum anxiety (global estimate of prevalence among mothers: 9.3%–18.0%, [33–36], and among fathers: 9.5% [33],) as the most common mental disorders are of special interest. However, also symptoms of somatization (i.e., physical symptoms like pain or fatigue), obsessive-compulsiveness (i.e., recurring intrusive thoughts such as contamination obsessions and/or repetitive behaviors such as washing), and anger/hostility (i.e., feelings of irritation, rage, and resentment up to aggressiveness) have been reported in the peripartum period [37–41]. These symptoms warrant attention as they occur more frequently during the peripartum period than in the general population and can cause serious suffering for those affected [37, 39]. To illustrate, postpartum intrusive thoughts or repetitive behaviors as well as feelings of irritation often focus on infant-related concerns (e.g., accidental harm to the infant), and can consequently be particularly shameful or distressing for affected parents [37]. Furthermore, when irritation escalates to aggressiveness, this may have serious consequences for involved family members [38]. In addition, structurally assessing somatization symptoms may be particularly crucial in the peripartum period, as they are important indicators of mental distress and might be overlooked or misattributed to normal pregnancy and postpartum physical changes [39, 40]. Including a broader spectrum of symptoms helps to identify individuals whose psychological distress may manifest differently from more common depression and anxiety measures. Importantly, parental mental health symptoms in the peripartum period can have long-term detrimental effects not only on the well-being of the affected individuals but also on the parent-infant relationship and the child’s behavioral and developmental outcomes [38, 42–45]. Consequently, a growing body of research has focused on identifying potential risk factors for poor mental health in the transition to parenthood.

### Adverse working conditions as risk factor for postpartum mental health symptoms

Previous investigations have focused on a variety of individual-level factors such as age, chronic/daily stress exposure, (past) stressful or traumatic life events (e.g., abuse, intimate partner violence), prior mental health disorders, obstetric experiences, difficult infant temperament, partnership conflict, and lack of social support as risk factors for postpartum mental health problems [46–50]. Ko et al. [51], for instance, linked stressful life events occurring within the year before birth to postpartum depression. However, studies on the role of *work factors as potential specific chronic/life stressors* are still lacking [18, 31]. As an example, a study examining the effects of chronic strains on postpartum-onset depression included measures of neighborhood safety, parenting strain, and relationship strain, but neglected strains related to employment, even though listing employment as a potential source of chronic strain in the introduction [50]. This is surprising, given that we already know from previous research (mainly *outside the peripartum period*) that the work environment is important for adult mental health [6, 52–57] and that (expectant) parents constitute a particularly vulnerable group [17, 45, 58, 59]. Importantly, the influence of working conditions does not diminish in the transition to parenthood: Across OECD countries, (expectant) mothers are entitled to around 18 weeks of maternity leave shortly before and after birth, remaining largely employed during pregnancy [60–62]; new fathers usually only take few months of parental leave *after* birth [63], and both have to plan and ensure the future compatibility of work and family life. In addition, the new financial responsibility reinforces the importance of a secure income.

According to the stress-associated Conservation of Resources Theory (COR; [64–66]), adverse working conditions can represent a threat to resources (e.g., personal resources such as skills, material resources such as tools for work, condition resources such as seniority, and energy resources such as positive emotions; [67, 68]) and, via increased attention to and appraisal of this threat, consume energy and lead to resource loss (e.g., negative emotions; [66]). This, in turn, reduces available resources to cope with upcoming strains – which are manifold within the transition to parenthood. In a cyclic process, this might lead to poor mental health in the long term. Specifically, in the context of transitioning to parenthood, COR implies that adverse prepartum working conditions reduce the parents’ resources and abilities to cope with the additional strains coming with this period of great changes and thereby might hinder a successful adjustment. Furthermore, this consideration suggests that adverse working conditions might contribute to a *general vulnerability* and thereby have an impact on overall mental health, including various mental health symptoms.

The limited evidence available suggests that working conditions *during the transition to parenthood* indeed have an important influence on postpartum mental health (e.g., [69]). For instance, precarious employment (including, e.g., temporary employment, low wages, and authoritarian treatment) during pregnancy predicted symptoms of depression in mothers eight weeks after childbirth [70]. In addition, higher psychosocial work stress (i.e., an imbalance of high effort and low reward at work) during the COVID-19 pandemic was associated with parental symptoms of depression and anger/hostility [38]. Furthermore, perceived pregnancy discrimination in the workplace was found to be indirectly related to maternal postpartum symptoms of depression via perceived stress during pregnancy [71]. In (expectant) fathers, poor job quality was identified as a significant risk factor for symptoms of psychological distress in the first year postpartum [72], and high job burden as well as low job satisfaction predicted birth-related posttraumatic stress disorder symptoms eight weeks postpartum [57].

Interestingly, certain aspects of the work environment appear to be particularly relevant to employees’ mental health, including abusive supervision (i.e., aggressive and hostile behavior by supervisors; [73, 74]), job insecurity (i.e., fear of losing one’s job or important features of it; [75–77]), and high job demand (i.e., high workload and time pressure experienced at work; [78–80]). First evidence from the peripartum period on these aspects suggests that experiencing *abusive supervision*, as well as vicarious abusive supervision (i.e., witnessing colleagues being abused) in the peripartum period can contribute to job-related emotional exhaustion and postpartum depressive symptoms in mothers [66]. On the contrary, supportive supervisor behaviors, defined as general feelings of emotional and instrumental support, can have a protective effect on parental mental health [81, 82]. Furthermore, cross-sectional studies report perceived *job insecurity* to be associated with a higher risk of depression and anxiety disorders in mothers [83, 84] and fathers [85] in the first year postpartum. In accordance, a more recent cross-sectional study conducted during the COVID-19 pandemic in the US found that perceived job insecurity was a significant predictor of symptoms of depression during the peripartum period [86]. Moreover, high *job demand* was linked to mental health problems in mothers and working-class dual-earner couples during the transition to parenthood [81, 87] and in lesbian and gay parents [88]. Supporting this, a Norwegian longitudinal study reported that prepartum work stress including high job demand predicted postpartum depression and anxiety in mothers [89]. In line with this, a recent systematic review on maternal return to work after giving birth identified total workload, which is part of the concept of job demand, as a significant predictor of maternal mental health [69]. Another recent longitudinal study suggests that the adverse working condition time urgency, which is also part of the concept of job demand, may even have both a direct and – via symptoms of postpartum depression and anxiety – indirect detrimental effect on maternal interactions with their child one year after birth [90]. Furthermore, some studies indicate that an increase in total workload (i.e., paid and unpaid work) is related to adverse postpartum mental health outcomes in mothers [91, 92].

Overall, research on the intersection of working conditions and mental health in the peripartum period is limited. The few available studies have mostly focused on mothers [85], whereas postpartum mental health issues are also experienced by fathers and female partners [93–95] – an issue that applies to almost all areas of peripartum mental health research [96, 97]. However, this is a critical gap to address within the work context, as, despite an increase in dual-earner families, it is still mainly mothers who reduce their working hours and take the majority of parental leave [98], while the working conditions of partners – with their potentially detrimental effects on mental health – remain constantly present during the transition to parenthood [14]. Interestingly, a recent study of our group found that only in fathers but not in mothers, lower job satisfaction and higher job burden during pregnancy serve as predictors of birth-related posttraumatic stress disorder symptoms eight weeks postpartum, highlighting the relevance of considering work-related factors in fathers’ postpartum mental health and suggesting that differential investigations of (expectant) mothers and partners could be informative [57]. A further shortcoming in the literature is that evidence mainly stems from cross-sectional studies. While these add valuable knowledge about associations at the time of assessment, it is crucial to consider the long-term effects of working conditions during the transition to parenthood, especially when at least one or both parents take parental leave. Moreover, many studies lack theory-based, validated measures of working conditions, resulting in a wide range of data that are difficult to compare with each other [53, 99, 100]. In addition, while former studies on work and mental health in the peripartum period mostly focused on depression and anxiety as outcomes (e.g., [101–103]), it should be acknowledged that individual reactions to potentially stressful working conditions might manifest themselves in a variety of mental health symptoms such as symptoms of somatization, obsessive-compulsiveness, and anger/hostility. To illustrate, some individuals may primarily exhibit somatic complaints, contamination or infant-related obsessions, or increased irritability rather than depressive or anxiety symptoms. Examining this broader symptom spectrum is crucial to identify and support individuals who might otherwise be overlooked. Finally, many studies on work and parental mental health analyze data gathered more than 20 years ago. In light of the current changes in the labor market, including digitalization and ongoing flexibilization (e.g., teleworking) – which have received another major boost due to the COVID-19 pandemic – and the associated impact on workers’ mental health [45, 104–107], it is imperative to update the results of older studies. In this context, it should also be noted that, due to the increase in dual-earner families [16], (expectant) parents nowadays represent an even more important and vulnerable group within the working population, firstly because of the growing proportion of working parents (especially due to more working mothers), and secondly because the challenge of reconciling work and family life is becoming increasingly prevalent as more and more parents take on a dual role.

### Aim of the present study

The aim of this study is to enhance our understanding of work-related risk factors for the adjustment of parents in the transition to parenthood. To address the identified research gaps, we will investigate the predictive value of adverse prepartum working conditions for a variety of mental health outcomes 14 months postpartum in both mothers and their partners. Based on the available literature and theoretical considerations, we hypothesize that higher levels of prepartum precarious employment, abusive supervision, job insecurity, and job demand will predict poorer mental health outcomes (i.e., more symptoms of depression, somatization, obsessive-compulsiveness, anxiety, and anger/hostility) 14 months postpartum in mothers and their male or female partner, controlling for relevant confounders (e.g., employment status, academic degree) and pre-existing symptoms of the respective mental health variable during pregnancy.

## Methods

### Study design

The study is based on data from the ongoing Dresden Study on Parenting, Work, and Mental Health (DREAM; “DResdner Studie zu Elternschaft, Arbeit und Mentaler Gesundheit”). DREAM is a prospective cohort study that examines the associations between parental work participation, role distribution, perinatal factors, stress factors, and long-term family (mental) health and intra-family relationships with currently seven measurement points from pregnancy (T1) to 7.5 years after childbirth (T7). Between June 2017 and the end of 2020, 3,860 expectant mothers and their male or female partners were recruited mainly at information events of obstetrical clinics in and around Dresden, Germany. Further information on the design, sample, and procedures can be found in the study protocol [108]. For the current analyses, questionnaire data of mothers and male and female partners on prepartum working conditions (T1) and mental health symptoms at 14 months postpartum (T3) were used.

### Sample

**Fig. 1:**
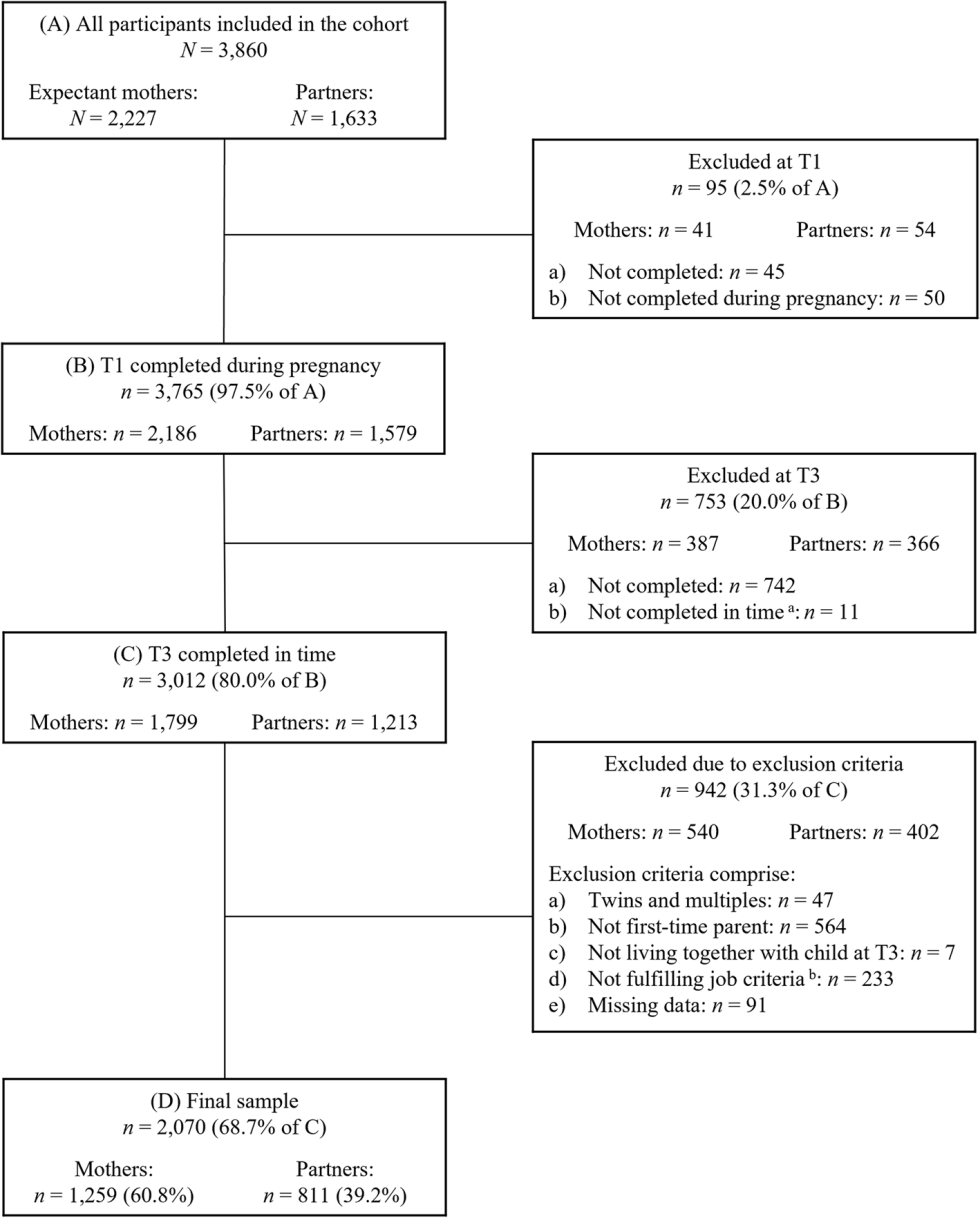
Flowchart of retention rate and exclusion criteria resulting in final sample *Note.* T1 = during pregnancy; T3 = around 14 months after the actual birth date. Data for the current study were extracted on March 10, 2023 (version 10 of the quality-assured data files; both T1 and T3 were already completed at the time of data extraction). ^a ^Not within 12 and 18 months after the actual birth date. ^b^ Job criteria leading to exclusion comprise attending school, working in an apprenticeship or voluntary service, being self-employed, and not being at least in marginal employment at T1 (self-rated)

By March 10th, 2023, *n* = 2,227 expectant mothers and *n* = 1,633 partners had answered the first questionnaire (T1). Of those, *n* = 1,799 mothers and *n* = 1,213 partners completed both the T1 and T3 questionnaire *in time* (i.e., T1 during pregnancy and T3 within 12–18 months after birth; see flow chart; Fig. 1). For the present study, inclusion criteria comprise 1) giving birth to a single child, 2) being a first-time parent, 3) still living together with the child 14 months after birth, 4) fulfilling relevant job criteria, i.e., being employed at least part-time or marginally at T1 excluding self-employment, 5) being at least 18 years old, and 6) providing data for relevant measures at both timepoints. The final sample consisted of 2,070 participants comprising 1,259 mothers (60.8%) and 811 male and female partners (39.2%).

### Measures

#### Adverse prepartum working conditions as predictors

As predictors, we included the working conditions precarious employment, abusive supervision, job insecurity, and job demand measured during pregnancy (T1). If participants were in pregnancy-related employment ban (see [109]) or had not worked since the pregnancy, participants were asked to answer the questions on working conditions referring to the 6 months prior to pregnancy or employment ban.

*Precarious employment* was measured with the German version of the Employment Precariousness Scale (EPRES; [110, 111]) that was specifically designed for epidemiological studies among waged workers. In previous investigations, it has shown good acceptability, internal consistency (Cronbach’s α ≥ 0.70), and construct validity [70, 111–113]. The instrument contains six subscales: *temporariness* (2 items), *disempowerment* (2 items), *vulnerability* (5 items), *wages* (3 items), *rights* (4 items), and *exercise rights* (6 items). The subscale disempowerment was excluded in this study due to many different types of employment within the sample. The overall EPRES score, ranging from 0 (*not precarious*) to 4 (*most precarious*), is the arithmetic mean of all subscale scores. In our sample, the internal consistency was acceptable (mothers: α = 0.74; partners: α = 0.72).

*Abusive supervision* was assessed with the EPRES subscale *vulnerability* [110, 111], which measures defenselessness to authoritarian treatment with five items answered on a 5-point frequency scale from 0 (*never*) to 4 (*always*). Example items are “*In relation to the way you are treated at work, can you tell me whether…You are defenseless towards unfair treatment by your superiors / … You are made to feel you can be easily replaced”* [110]. The items are similar to those used in other studies that identified abusive supervision as relevant for workers’ depression and emotional exhaustion [78]. Total abusive supervision scores are computed as simple averages resulting in a 0–4 range with higher scores indicating more abusive supervision. Internal consistency was acceptable (mothers: α = 0.78; partners: α = 0.73) in the current study.

*Job insecurity* and *job demand* were measured via two items of the German version of the Effort-Reward Imbalance Questionnaire (ERI; [114, 115]), respectively. Job insecurity has previously been operationalized this way by Marchand et al. [78]. An example item is “*I have experienced or I expect to experience an undesirable change in my work situation*”. The two items measuring job demand were selected based on the similarity to items used for this construct in prior studies [81, 116]. An example item is “*I have constant time pressure due to a heavy work load”*. Responses are based on a 4-point Likert scale from 1 (*strongly disagree*) to 4 (*strongly agree),* leading to total sum scores ranging from 2–8 with higher scores indicating more job insecurity/job demand. Internal consistency, measured with the Spearman-Brown coefficient ρ as recommended for two-item scales [117], was acceptable for the two items selected for job insecurity (mothers ρ = 0.61; partners ρ = 0.65) and job demand (mothers ρ = 0.65; partners ρ = 0.61).

#### Postpartum mental health outcomes

Mental health outcomes were measured at 14 months postpartum (T3) and comprised symptoms of depression, somatization, obsessive-compulsiveness, anxiety, and anger/hostility.

*Symptoms of depression* were measured by the validated German version of the Edinburgh Postnatal Depression Scale (EPDS; [118]). The EPDS is the gold standard for assessing symptoms of depression throughout the peripartum period in mothers as well as partners [119–121]. The 10 self-report items assess symptom severity in the last seven days. Answers were given on a 4-point scale (0–3), resulting in a sum score ranging from 0–30 with higher scores indicating higher levels of depressive symptoms. Internal consistency was good in the current study (mothers: α = 0.83; partners: α = 0.81).

*Symptoms of somatization, obsessive-compulsiveness, anxiety,* and *anger/hostility* were measured with the corresponding subscales of the validated German version of the Symptom-Checklist Revised (SCL-90-R; [122]), including 12, 10, 10, and 6 self-report items, respectively. Answers refer to symptoms experienced within the last seven days and were given on a 5-point Likert scale from 0 (*not at all*) to 4 (*very strong*) resulting in scale-length-dependent sum score ranges of 0–48, 0–40, 0–40, and 0–24, respectively, with higher scores indicating more symptom severity. The internal consistency of all subscales was acceptable or good among mothers and partners (0.73 ≤ α ≤ 0.83).

#### Potential confounding variables

We considered the following variables as potential confounders because associations with the predictor or outcome variables were reported in previous studies: Employment status, academic degree, age, duration of parental leave, and COVID-19 pandemic exposure. *Employment status* was measured via items on full-time, part-time, and marginal employment, while multiple answers were possible. Employment status was subsequently dichotomized by combining part-time and marginal employment (0 = *full-time*; 1 = *part-time or marginal*). If participants chose more than one category, the more time-consuming option was recorded. *Academic degree* was assessed at T1 with a question based on the German National Cohort Consortium [123] and was subsequently dichotomized as 1 (*academic degree*) or 0 (*no academic degree*). *Age* was measured in years at T1 and *duration of parental leave* was assessed in months at T3 (i.e., the duration relates to the parental leave taken up to T3). *COVID-19 pandemic exposure* was considered as a potential confounder to account for possible effects on mental health [124, 125]. Participants were grouped into two categories based on the date of completion at T3 (i.e., when the outcome variable was assessed). Those who completed T3 between March 10th, 2020, and January 15th, 2023 were assigned to the “during pandemic” group, otherwise, they were placed in the “before/after pandemic” group. Finally, we controlled for *pre-existing (T1) symptoms of the respective mental health variable*, i.e., symptoms of depression at T1 with the EPDS, symptoms of somatization, obsessive-compulsiveness, anxiety, and anger/hostility at T1 with the corresponding subscale of the SCL-90-R.

### Statistical analyses

Analyses were performed using SPSS 27 [126]. Participant’s mean values replaced missing items if they filled in at least 80% of the items from psychometric scales. We conducted descriptive analyses for sociodemographic characteristics of the sample and the primary study variables (*N*, rates in %, *M*, *SD*). Furthermore, attrition analyses were conducted for sociodemographic characteristics, predictors, outcomes, and potential confounders, comparing participants who completed T1 and T3 to participants who only completed T1 (see Additional file 1). Potential confounding variables were identified based on theoretical considerations and those significantly correlating with at least one of the mental health outcomes in preliminary correlation analyses (see Additional file 2) were included as confounders in regression analyses (separately for mothers and partners). In the main analyses, Pearson correlations for all study variables were calculated and hierarchical multiple regression analyses were conducted to examine prospective associations between prepartum working conditions and mental health outcomes 14 months postpartum. We calculated separate regression models for each mental health outcome. Regarding working conditions, precarious employment, which reflects a broader, multidimensional construct [53] was investigated separately as predictor to examine its contribution to the outcome among confounders. The more specific adverse working conditions abusive supervision, job insecurity, and job demand were included together as predictors to see if they could explain separate parts of the variance in mental health. In all models, working conditions and sociodemographic confounders were included in the first step to evaluate their contributions to the outcome (Model 1), before entering pre-existing (T1) symptoms of the respective mental health variable in the second step to determine whether working conditions predict the mental health outcome beyond prior symptomatology (Model 2). The Mahalanobis distance was used to identify multivariate outliers considering all relevant study variables [127]. Following, sensitivity analyses for the influence of possible multivariate outliers were conducted (i.e., main analyses were calculated with versus without outliers; see Additional file 4). Due to residuals showing non-normal distributions, bootstrap sampling procedures with 5,000 iterations were applied. For mothers and partners, all analyses were performed separately. A two-tailed *p*-value < 0.05 was considered significant. To address multiple testing, we applied the Šidák correction $(1-{\left(1-\alpha \right)}^\frac{1}{m})$, controlling the familywise error rate across our five predicted mental health outcomes (*m* = 5), resulting in an adjusted significance threshold of *p* < 0.01. To ensure transparency, we report unadjusted *p*-values and indicate which findings remain significant after applying the adjusted significance threshold.

## Results

### Sample characteristics

The sample characteristics and descriptive statistics for working conditions and mental health outcomes are provided in Table 1. Compared to the average population of Dresden [128, 129], the sample consisted mainly of parents with rather high education (58.9% of the mothers and 58.6% of the partners holding an academic degree) and contained fewer inhabitants with birth countries other than Germany (3.2% of the mothers and 1.7% of the partners born in another country than Germany). Of the expectant mothers, 29.0% were in employment ban at T1. Therefore, their answers to working conditions were filled in retrospectively for the 6 months before pregnancy/employment ban. The majority of mothers (77.8%) and partners (90.4%) worked full-time at T1/prior to employment ban. On average, mothers took longer parental leave than partners (12.7 ± 2.00 months versus 3.35 ± 2.69 months).

**Table 1:** Descriptive statistics for mothers and partners

**Characteristic**	**Mothers** *n *^a^ = 1,259	**Partners** *n *^a^ = 811
	*M* ± *SD* (Range) or *n* (%) ^b^	*M* ± *SD* (Range) or *n* (%) ^b^
**Age (years)**	29.9 ± 3.60 (20–43)	32.0 ± 4.36 (21–54)
**Sex**		
Male	-	799 (98.5)
Female	-	12 (1.5)
**Gestational week at T1**	29.0 ± 6.43 (8–41)	29.4 ± 6.62 (8–41)
**Age of child in months at T3**	13.8 ± 0.60 (12–18)	13.9 ± 0.58 (12–18)
**County of birth**		
Germany	1,215 (96.8)	793 (98.3)
Other	40 (3.2)	14 (1.7)
**Relationship status**		
Permanent relationship	1,239 (89.4)	809 (99.9)
No permanent relationship	20 (1.6)	1 (0.1)
**Academic degree**		
Academic degree	738 (58.9)	470 (58.6)
No academic degree	516 (41.1)	332 (41.4)
**Employment status at T1 **^c^		
Full-time employment	979 (77.8)	733 (90.4)
Part-time employment	237 (18.8)	56 (6.9)
Marginal employment	43 (3.4)	22 (2.7)
**Working hours per week at T1 **^c^	38.6 ± 7.79 (3–70)	40.7 ± 7.32 (4–70)
**Monthly income of main job at T1 **^c,d^		
More than 2,500€	186 (14.8)	200 (24.7)
1,501–2,500€	759 (60.4)	482 (59.5)
851–1,500€	255 (20.3)	98 (12.1)
451–850€	21 (1.7)	10 (1.2)
Less than 450€	35 (2.8)	20 (2.5)
**Employment ban at T1**		
Yes	364 (29.0)	-
No	891 (71.0)	-
**Duration of parental leave until T3 (in months)**	12.7 ± 2.00 (0–18)	3.35 ± 2.69 (0–18)
**T1 Working conditions**		
Precarious employment ^e^ (possible range 0–4)	0.95 ± 0.44 (0–2.86)	0.77 ± 0.40 (0–2.38)
Abusive supervision ^f^ (possible range 0–4)	0.78 ± 0.72 (0–4)	0.55 ± 0.59 (0–3.6)
Job insecurity ^g^ (possible range 2–8)	3.73 ± 1.37 (2–8)	3.31 ± 1.22 (2–8)
Job demand ^g^ (possible range 2–8)	5.87 ± 1.39 (2–8)	5.78 ± 1.29 (2–8)
**T3 Symptoms of**		
Depression ^h^ (possible range 0–30)	5.47 ± 4.12 (0–27)	3.97 ± 3.55 (0–19)
Somatization ^i^ (possible range 0–48)	3.38 ± 3.60 (0–38)	2.51 ± 3.01 (0–25)
Obsessive-compulsiveness ^i^ (possible range 0–40)	4.06 ± 4.18 (0–29)	3.33 ± 3.81 (0–25)
Anxiety ^i^ (possible range 0–40)	1.80 ± 2.75 (0–28)	1.26 ± 2.12 (0–20)
Anger/hostility ^i^ (possible range 0–24)	2.21 ± 2.60 (0–20)	1.58 ± 2.20 (0–18)

^a ^*n* varies slightly due to missing data of some participants. ^b ^Valid percent. Percentages may not sum to 100% due to rounding. ^c ^If mothers were in employment ban at T1, employment status/working hours before employment ban was used. ^d ^Income after taxes. ^e ^Measured with the Employment Precariousness Scale (EPRES). ^f ^Measured with the EPRES subscale vulnerability. ^g ^Measured with two items of the Effort-Reward Imbalance Questionnaire (ERI). ^h ^Measured with the Edinburgh Postnatal Depression Scale (EPDS). ^i ^Measured with the corresponding subscale of the Symptom Checklist-90-Revised (SCL-90-R)

### Preliminary analyses

#### Attrition analyses and rates of missing data in the final sample

Attrition analyses were conducted for sociodemographic characteristics, working conditions, mental health variables, and potential confounders, comparing participants who completed T1 and T3 to participants who only completed T1 (see Additional file 1). In both mothers and partners, completers were significantly less precariously employed and among partners, completers were significantly less exposed to abusive supervision. Concerning mental health, completers reported significantly fewer symptoms of depression and anger/hostility during pregnancy in both mothers and partners, as well as fewer symptoms of obsessive-compulsiveness in mothers. Completers in both mothers and partners were significantly more often holding an academic degree and were less often born in another country than Germany. For both mothers and partners there were no significant differences between completers and non-completers regarding any other variables. Rates of missing data in the final sample (*N* = 2,070) were very low, with less than 1% of items missing per scale (range 0.25–0.88%; see Table S3 in Additional file 1).

#### Confounding variables

Of the sociodemographic confounders, age, duration of parental leave, academic degree, and employment status were selected for regression analyses of mothers, and academic degree and employment status for regression analysis of partners (for preliminary correlational analyses see Additional file 2). COVID-19 pandemic exposure was not significantly associated with mental health outcomes in neither mothers nor partners and was therefore not included in regression analyses.

#### Sensitivity analyses

After identifying *n* = 67 multivariate outliers among mothers and *n* = 46 among partners using Mahalanobis distance considering all relevant study variables, sensitivity analyses for the influence of possible multivariate outliers were performed (i.e., regression analyses were calculated with versus without outliers). As the sensitivity analyses indicated different significances in at least one of the calculated regression analyses, the final correlation (see Table 2) and regression analyses (see Tables 3 and 4) are reported excluding these outliers, as the results without outliers are assumed to provide more accurate estimates. However, results of correlation and regression analyses with outliers included are provided in Additional file 4.

**Table 2:** Correlation matrix of study variables of mothers (above diagonal) and partners (below diagonal) after exclusion of multivariate outliers

Variable		1	2	3	4	5	6	7	8	9	10	11	12	13	14	15	16	17	18
1)	**Precarious employment T1 **(EPRES)	-	.57^**^	.36^**^	.09^**^	.14^**^	.16^**^	.17^**^	.17^**^	.13^**^	−.26^**^	−.06^*^	−.18^**^	.22^**^	.20^**^	.16^**^	.19^**^	.14^**^	.12^**^
2)	**Abusive supervision T1** (EPRES subscale)	.42^**^	-	.42^**^	.31^**^	.22^**^	.20^**^	.21^**^	.23^**^	.17^**^	−.06*	.11^**^	−.15^**^	−.05	.27^**^	.23^**^	.28^**^	.25^**^	.21^**^
3)	**Job insecurity T1** (ERI selected items)	.23^**^	.35^**^	-	.16^**^	.16^**^	.14^**^	.13^**^	.14^**^	.10^**^	.04	.04	−.05	−.01	.20^**^	.17^**^	.18^**^	.15^**^	.09^**^
4)	**Job demand T1** (ERI selected items)	−.03	.21^**^	.17^**^	-	.08^**^	.14^**^	.09^**^	.12^**^	.07^*^	.06^*^	.06^*^	−.13^**^	−.20^**^	.12^**^	.11^**^	.11^**^	.11^**^	.04
5)	**Symptoms of depression T3** (EPDS)	.12^**^	.26^**^	.19^**^	.05	-	.43^**^	.56^**^	.53^**^	.56^**^	−.07^*^	−.00	−.04	.04	.50^**^	.32^**^	.38^**^	.35^**^	.29^**^
6)	**Symptoms of somatization T3 **^a^	.11^**^	.19^**^	.06	.05	.38^**^	-	.44^**^	.44^**^	.39^**^	−.04	−.01	−.05	.01	.31^**^	.46^**^	.35^**^	.32^**^	.23^**^
7)	**Symptoms of obsessive-compulsiveness T3 **^a^	.14^**^	.26^**^	.11^**^	.00	.57^**^	.47^**^	-	.59^**^	.57^**^	−.03	−.00	−.01	.01	.35^**^	.28^**^	.49^**^	.34^**^	.25^**^
8)	**Symptoms of anxiety T3 **^a^	.11^**^	.25^**^	.10^**^	.02	.50^**^	.44^**^	.62^**^	-	.46^**^	−.05	−.02	−.01	.06^*^	.33^**^	.29^**^	.35^**^	.42^**^	.22^**^
9)	**Symptoms of anger/hostility T3 **^a^	.09^*^	.24^**^	.08^*^	.05	.48^**^	.37^**^	.49^**^	.50^**^	-	−.06^*^	−.01	−.02	.01	.32^**^	.29^**^	.35^**^	.29^**^	.42^**^
10)	**Age**	−.26^**^	−.06	.09^*^	.11^**^	−.02	−.06	−.03	−.00	−.03	-	.06^*^	.23^**^	−.10^**^	−.07^*^	−.11^**^	−.09^**^	−.03	−.11^**^
11)	**Duration of parental leave up to T3**	.04	.01	−.01	−.09^*^	−.01	.00	−.04	−.07	.01	-.02	-	.01	.01	−.02	.01	.05	.04	.01
12)	**Academic degree **^b^	−.05	−.13^**^	.00	−.04	−.00	−.09^*^	.03	.01	−.04	.04	.05	-	−.01	−.11^**^	−.11^**^	−.06	−.04	−.04
13)	**Employment status T1 **^c^	.32^**^	−.06	.04	−.16^**^	.11^**^	.05	.07	.03	.02	−.17^**^	.04	.05	-	.04	.01	.01	.01	.02
14)	**Symptoms of depression T1** (EPDS)	.12^**^	.30^**^	.25^**^	.14^**^	.52^**^	.29^**^	.37^**^	.37^**^	.30^**^	−.03	−.02	.00	.02	-	.36^**^	.52^**^	.52^**^	.49^**^
15)	**Symptoms of somatization T1 **^a^	.09^*^	.23^**^	.10^**^	.11^**^	.28^**^	.50^**^	.31^**^	.32^**^	.27^**^	−.07^*^	.01	−.08^*^	.03	.37^**^	-	.51^**^	.46^**^	.37^**^
16)	**Symptoms of obsessive-compulsiveness T1 **^a^	.16^**^	.31^**^	.20^**^	.05	.37^**^	.30^**^	.59^**^	.41^**^	.33^**^	−.02	−.01	.03	.06	.54^**^	.47^**^	-	.59^**^	.51^**^
17)	**Symptoms of anxiety T1 **^a^	.10^**^	.27^**^	.17^**^	.13^**^	.38^**^	.35^**^	.43^**^	.51^**^	.34^**^	.01	−.04	.05	.02	.54^**^	.50^**^	.59^**^	-	.45^**^
18)	**Symptoms of anger/hostility T1 **^a^	.09^*^	.30^**^	.19^**^	.13^**^	.31^**^	.27^**^	.33^**^	.36^**^	.47^**^	−.00	−.03	−.03	−.02	.45^**^	.37^**^	.52^**^	.48^**^	-

*Note*. Pearson correlations for mothers (*n* ranges from 1,047 to 1,187 due to missing data) are shown above the diagonal. Pearson correlations for partners (*n* ranges from 598 to 763 due to missing data) are shown below the diagonal. *EPRES* Employment Precariousness Scale, *ERI* Effort-Reward Imbalance Questionnaire, *EPDS* Edinburgh Postnatal Depression Scale

^*^p < .05; ^**^p < .01

^a ^Measured with subscales of the Symptom Checklist-90-Revised (SCL-90-R). ^b ^0 = no academic degree and 1 = academic degree. ^c ^0 = full-time, 1 = part-time or marginal; if mothers were in employment ban at T1, employment status before employment ban was used

**Table 3:** Hierarchical multiple regression analyses for mothers after exclusion of multivariate outliers

Outcomes	Predictors	Model 1					Model 2				
Symptoms of		**B**	**BCa 95% CI**	**β**	***p***	***R***^**2**^	**B**	**BCa 95% CI**	**β**	***p***	***R***^**2**^
**Depression**	Precarious employment ^a^	1.30	[0.73,1.88]	.14	** < .001**	.02	0.45	[−0.06,0.98]	.05	*.089*	.25
	Abusive supervision	0.99	[0.61,1.39]	.18	** < .001**		0.49	[0.14,0.83]	.09	**.004**	
	Job insecurity	0.29	[0.10,0.49]	.10	**.002**		0.12	[−0.05,0.30]	.04	*.161*	
	Job demand	0.05	[−0.12,0.22]	.02	.577	.06	−0.02	[−016,0.13]	−.01	.794	.27
**Somatization**	Precarious employment ^a^	1.04	[0.60,1.49]	.15	** < .001**	.02	0.64	[0.23,1.06]	.09	**.004**	.20
	Abusive supervision	0.59	[0.29,0.89]	.14	** < .001**		0.28	[0.02,0.56]	.07	*.042*	
	Job insecurity	0.17	[0.03,0.32]	.08	.021		0.08	[−0.06,0.21]	.03	*.299*	
	Job demand	0.19	[0.06,0.31]	.09	**.004**	.05	0.14	[0.02,0.25]	.07	.017	.21
**Obsessive-compulsiveness**	Precarious employment ^a^	1.54	[1.00,2.10]	.18	** < .001**	.03	0.73	[0.22,1.24]	.08	**.005**	.26
	Abusive supervision	1.02	[0.64,1.40]	.20	** < .001**		0.46	[0.12,0.81]	.09	***.009***	
	Job insecurity	0.18	[0.00,0.35]	.07	.046		0.07	[−0.09,0.24]	.03	.366	
	Job demand	0.05	[−0.11,0.20]	.02	.587	.05	0.01	[−0.13,0.15]	.00	.924	.25
**Anxiety**	Precarious employment ^a^	0.76	[0.42,1.12]	.14	** < .001**	.03	0.45	[0.13,0.79]	.09	**.009**	.18
	Abusive supervision	0.57	[0.34,0.82]	.19	** < .001**		0.32	[0.12,0.54]	.10	**.005**	
	Job insecurity	0.08	[−0.02,0.19]	.05	*.126*		0.04	[−0.06,0.15]	.03	.430	
	Job demand	0.10	[0.01,0.19]	.07	.037	.06	0.07	[−0.02,0.15]	.04	.135	.19
**Anger/hostility**	Precarious employment ^a^	0.74	[0.42,1.06]	.14	** < .001**	.02	0.45	[0.17,0.75]	.09	**.003**	.19
	Abusive supervision	0.56	[0.32,0.81]	.17	** < .001**		0.26	[0.03,0.48]	.08	.026	
	Job insecurity	0.06	[−0.06,0.19]	.04	.300		0.05	[−0.06,0.16]	.03	.329	
	Job demand	0.04	[−0.06,0.14]	.02	*.445*	.04	0.04	[−0.06,0.14]	.02	.404	.21

*Note*. Results of multiple regression analyses with working conditions and sociodemographic confounders age, duration of parental leave, academic degree, and employment status (Model 1) and pre-existing (T1) symptoms of respective mental health variable added in the second step (Model 2). *R*^2^ = adjusted. Working conditions were measured during pregnancy (T1) and mental health outcomes 14 months after childbirth (T3). For standardized βs of confounding variables see Additional file 3. BCa 95% CI = Bias-corrected and accelerated 95% bootstrap confidence interval

*p*-values and BCa CI based on 5,000 bootstrap samples. Significant *p*-values after applying the Šidák correction (*p* < .01) are presented in bold. Significances deviating from analyses with multivariate outliers included (see Additional file 4) are presented in italics

^a ^Precarious employment was analyzed in a separate regression model

**Table 4:** Hierarchical multiple regression analyses for partners after exclusion of multivariate outliers

Outcomes	Predictors	Model 1					Model 2				
Symptoms of		**B**	**BCa 95% CI**	**β**	***p***	***R***^**2**^	**B**	**BCa 95% CI**	**β**	***p***	***R***^**2**^
**Depression**	Precarious employment ^a^	0.81	[0.18,1.47]	.10	.015	.01	0.30	[−0.27,0.87]	.04	.306	.25
	Abusive supervision	1.42	[0.94,1.93]	.24	** < .001**		0.74	[0.32,1.17]	.12	** < .001**	
	Job insecurity	0.27	[0.07,0.47]	.10	.013		0.06	[−0.12,0.25]	.02	.556	
	Job demand	0.01	[−0.17,0.19]	.00	.953	.09	−0.08	[−0.24,0.12]	−.03	.370	.29
**Somatization**	Precarious employment ^a^	0.69	[0.18,1.23]	.12	.012	.01	0.46	[0.00,0.93]	.08	.062	.23
	Abusive supervision	0.85	[0.44,1.28]	.20	** < .001**		0.39	[0.05,0.75]	.09	*.025*	
	Job insecurity	0.00	[−0.15,0.16]	.00	.994		−0.01	[−0.14,0.13]	−.00	.927	
	Job demand	0.04	[−0.10,0.17]	.02	*.586*	.04	−0.02	[−0.14,0.13]	−.01	.778	.26
**Obsessive-compulsiveness**	Precarious employment ^a^	1.18	[0.57,1.83]	.15	** < .001**	.02	0.55	[0.03,1.09]	.07	.046	.28
	Abusive supervision	1.62	[1.12,2.14]	.28	** < .001**		0.63	[0.19,1.08]	.11	**.004**	
	Job insecurity	0.04	[−0.15,0.22]	.01	.708		−0.11	[−0.28,0.06]	−.04	.195	
	Job demand	−0.12	[−0.30,0.06]	−.05	.213	.07	−0.08	[−0.24,0.08]	−.03	.336	.34
**Anxiety**	Precarious employment ^a^	0.51	[0.20,0.85]	.12	**.002**	.01	0.29	[0.02,0.59]	.07	.045	.22
	Abusive supervision	0.87	[0.59,1.20]	.27	** < .001**		0.46	[0.21,0.72]	.14	** < .001**	
	Job insecurity	0.03	[−0.08,0.14]	.02	.597		−0.02	[−0.14,0.09]	−.02	.663	
	Job demand	−0.04	[−0.15,0.06]	−.03	.447	.07	−0.08	[−0.17,0.01]	−.06	.094	.28
**Anger/hostility**	Precarious employment ^a^	0.38	[0.06,0.70]	.09	.021	.00	0.15	[−0.13,0.44]	.04	.313	.22
	Abusive supervision	0.70	[0.42,0.98]	.24	** < .001**		0.35	[0.09,0.60]	.12	**.007**	
	Job insecurity	−0.01	[−0.13,0.11]	−.01	.863		−0.06	[−0.17,0.06]	−.04	.304	
	Job demand	−0.02	[−0.12,0.08]	−.01	.741	.05	−0.04	[−0.13,0.04]	−.04	.300	.22

*Note*. Results of multiple regression analyses with working conditions and sociodemographic confounders academic degree and employment status (Model 1) and pre-existing (T1) symptoms of respective mental health variable added in the second step (Model 2). *R*^2^ = adjusted. Working conditions were measured during pregnancy (T1) and mental health outcomes 14 months after childbirth (T3). For standardized βs of confounding variables see Additional file 3. BCa 95% CI = Bias-corrected and accelerated 95% bootstrap confidence interval

*p*-values and BCa CI based on 5,000 bootstrap samples. Significant *p*-values after applying the Šidák correction (*p* < .01) are presented in bold. Significances deviating from analyses with multivariate outliers included (see Additional file 4) are presented in italics

^a^ Precarious employment was analyzed in a separate regression model

### Main analyses

#### Precarious employment as a predictor of mental health symptoms

Precarious employment during pregnancy significantly correlated with all mental health outcomes 14 months postpartum in mothers (*p*s < 0.001) and partners (*p*s < 0.05; see Table 2). In hierarchical regression analyses controlling for relevant sociodemographic confounders (Model 1), precarious employment was a significant predictor for all mental health outcomes in mothers (βs = 0.14 to 0.18, *ps* < 0.001, Table 3) and partners (βs = 0.09 to 0.15, *p*s < 0.05, Table 4). Whereas all regression coefficients remained significant after Šidák correction in mothers, this was only the case for symptoms of obsessive-compulsiveness and anxiety in partners (*p* < 0.001, *p* = 0.002, respectively).

After controlling for pre-existing symptoms of the respective mental health outcome at T1 in addition to sociodemographic confounders (Model 2), precarious employment remained a significant predictor for symptoms of somatization (β = 0.09, *p* = 0.004), obsessive-compulsiveness (β = 0.08, *p* = 0.005), anxiety (β = 0.09, *p* = 0.009), and anger/hostility (β = 0.09, *p* = 0.003), all significant at Šidák-adjusted level, but not for symptoms of depression (β = 0.05, *p* = 0.089) in mothers. In partners, precarious employment remained a significant predictor for symptoms of obsessive-compulsiveness (β = 0.07, *p* = 0.046) and anxiety (β = 0.07, *p* = 0.045), although not below the threshold according to the Šidák correction; it also no longer significantly predicted the other mental health outcomes in partners after controlling for their respective pre-existing symptomatology (Model 2; βs = 0.04 to 0.08, *ps* > 0.062).

#### Abusive supervision as a predictor of mental health symptoms

Abusive supervision during pregnancy correlated significantly with all mental health outcomes 14 months postpartum in mothers and partners (*ps* < 0.001, see Table 2). In hierarchical regression analyses controlling for relevant sociodemographic confounders (Model 1), abusive supervision was a significant predictor for all mental health outcomes in mothers (βs = 0.14 to 0.20, *ps* < 0.001, Table 3) and partners (βs = 0.20 to 0.28, *ps* < 0.001, Table 4), all significant at Šidák-adjusted level.

After controlling for pre-existing symptoms of the respective mental health outcome at T1 in addition to sociodemographic confounders (Model 2), abusive supervision remained a significant predictor for all mental health outcomes in mothers (βs = 0.07 to 0.10, *ps* < 0.05) and partners (βs = 0.09 to 0.14, *ps* < 0.05). At the Šidák-adjusted level, the regression coefficients remained significant for symptoms of depression, obsessive-compulsiveness, and anxiety in mothers (*p* = 0.004, 0.009, 0.005, respectively) and symptoms of depression, obsessive-compulsiveness, anxiety, and anger/hostility in partners (*p* < 0.001, *p* = 0.004, *p* < 0.001, *p* = 0.007, respectively).

#### Job insecurity as a predictor of mental health symptoms

Job insecurity during pregnancy correlated significantly with all mental health outcomes 14 months postpartum in mothers (*p*s < 0.001) and all mental health outcomes (*p*s < 0.05) besides symptoms of somatization (*p* = 0.099) in partners (see Table 2). In hierarchical regression analyses controlling for relevant sociodemographic confounders (Model 1), job insecurity was a significant predictor for symptoms of depression, somatization, and obsessive-compulsiveness in mothers (βs = 0.07 to 0.10, *ps* < 0.05, Table 3) and symptoms of depression in partners (β = 0.10, *p* = 0.013, Table 4), but not for the remaining mental health outcomes (βs = −0.01 to 0.05, *ps* > 0.126). When applying the Šidák-adjusted significance threshold of *p* < 0.01, only the regression coefficient for symptoms of depression in mothers (*p* = 0.002) remained significant.

In both mothers and partners, job insecurity no longer significantly predicted any of the mental health outcomes after controlling for the respective pre-existing symptomatology in addition to sociodemographic confounders (Model 2; βs = −0.04 to 0.04, *ps* > 0.161).

#### Job demand as a predictor of mental health symptoms

Job demand during pregnancy correlated significantly with all mental health outcomes 14 months postpartum in mothers (*p*s < 0.05), but none of the mental health outcomes in partners (*p*s > 0.140, see Table 2). In hierarchical regression analyses controlling for relevant sociodemographic confounders (Model 1), job demand was a significant predictor for symptoms of somatization and anxiety in mothers (βs = 0.09 and 0.07, respectively*, p*s < 0.05, Table 3) but did not predict the remaining mental health outcomes in mothers (βs = 0.02, *ps* > 0.445) or any of the mental health outcomes in partners (βs = −0.05 to 0.02, *ps* > 0.213, Table 4). Thereby, only the predictive relationship with symptoms of somatization in mothers (*p* = 0.004) remained significant after Šidák correction.

After controlling for pre-existing symptoms of the respective mental health outcome at T1 in addition to sociodemographic confounders (Model 2), job demand remained a significant predictor for symptoms of somatization (β = 0.07, *p* = 0.017, non-significant at Šidák-adjusted level), but not for symptoms of anxiety (β = 0.04, *p* = 0.135) in mothers. In both mothers and partners, job demand did not predict any of the (remaining) mental health outcomes after controlling for their respective pre-existing symptomatology (βs = −0.06 to 0.02, *ps* > 0.094).

## Discussion

The present study aimed to enhance our understanding of work-related risk factors for the adjustment of parents in the transition to parenthood by investigating the predictive value of adverse prepartum working conditions for a variety of mental health outcomes 14 months postpartum in both mothers and their partners. Our findings provide evidence for the predictive role of adverse prepartum working conditions (i.e., precarious employment and exposure to abusive supervision, job insecurity, and job demand) for parental mental health (i.e., symptoms of depression, somatization, obsessive-compulsiveness, anxiety, and anger/hostility) 14 months after childbirth when controlling for relevant sociodemographic confounders and symptom levels during pregnancy. Precarious employment and abusive supervision emerged as particularly stable predictors of mental health outcomes, whereas results for the predictive value of job insecurity and job demand were mixed.

*Precarious employment* significantly predicted all mental health outcomes in mothers and partners, supporting the results from recent systematic/scoping reviews and a meta-analysis [53, 55, 130] that identified precarious employment as an important risk factor for mental health symptoms *in diverse worker samples*. Moreover, this finding is in line with a recent study by our group that identified precarious employment *during pregnancy* as a significant predictor of symptoms of depression in mothers eight weeks after childbirth [70]. After controlling for symptoms of the respective mental health variable during pregnancy in our regression analyses, precarious employment remained a significant predictor for mothers’ and partners’ symptoms of obsessive-compulsiveness and anxiety, as well as mothers’ symptoms of somatization and anger/hostility. However, it no longer predicted symptoms of depression in mothers and partners and symptoms of somatization and anger/hostility in partners. Whereas all significant findings survived Šidák correction in mothers, in partners this was only the case for symptoms of obsessive-compulsiveness and anxiety before controlling for prior mental health symptoms. Together, our findings indicate that precarious employment may be crucial for parental, especially maternal, postpartum mental health – for several symptoms independent of pre-existing symptomatology, particularly anxiety-related symptoms. Given that precarious employment is a multidimensional construct encompassing a range of unfavorable objective (e.g., temporary employment, low wages) and subjective (e.g., supervisor behavior) working conditions [53], the prospective associations we found with postpartum mental health point to the necessity of considering diverse aspects of the work environment to gain a comprehensive understanding of their impact on new parents.

*Abusive supervision* significantly predicted all mental health outcomes in mothers and partners, even after controlling for symptoms of the respective mental health variable during pregnancy. When using Šidák correction, all predictive relationships remained significant *before* controlling for prior symptoms; *after* controlling for prior symptoms, all predictive relationships except for symptoms of somatization (in both mothers and partners) and anger/hostility (in mothers) remained significant. Together, these findings are in line with former research that identified abusive supervision as a risk factor for workers’ mental health *outside the peripartum period* [78, 131, 132]. Furthermore, it is consistent with previous investigations showing that supportive supervisor behaviors can have a protective effect on parental mental health [81, 82], assuming that supportive supervision can be understood as the opposite or absence of abusive supervision. Former research emphasizes that abusive supervision violates the important moral principle of treating people respectfully and can evoke several negative stress-related emotions in employees, such as anger, fear, and shame [133], which may explain the particular detrimental effect on postpartum mental health that we found in our sample. Interestingly, a recent study on mothers during the transition to parenthood found that even *witnessing* abusive supervision predicted adverse mental health outcomes, beyond their own experience [66], again highlighting the particularly stressful nature of abusive supervision.

*Job insecurity* significantly predicted symptoms of depression in mothers and partners as well as symptoms of somatization and obsessive-compulsiveness in mothers, which is in line with results from the general working population [76, 77], working parents [45, 58, 134], and former research identifying job insecurity as a risk factor for parental depression and anxiety symptoms in the first year postpartum [83–85]. However, only the predictive relationship with symptoms of depression in mothers survived Šidák correction. Furthermore, after controlling for the respective symptom levels during pregnancy, none of the regression coefficients remained significant in our sample, indicating no additive predictive value above the previous mental health status. Moreover, job insecurity did not predict any of the other mental health outcomes in mothers and partners. These findings appear to contradict the aforementioned studies. However, it should be noted that the cited studies on new parents were cross-sectional and did not include outcomes other than depression and anxiety – an important difference from our study design. Furthermore, a recent meta-analysis, which included only prospective cohort studies, reported inconsistent associations between job insecurity and stress-related mental disorders [6], which is in line with our findings that associations depend on the outcome and may not explain variance beyond the preceding symptomatology. Another reason for our mixed results compared to international studies may be that workers in Germany generally enjoy a comparatively high level of security and can mostly rely on regulations on unemployment benefits after job loss, and therefore may perceive job insecurity as less threatening. Furthermore, at least mothers in our sample can mostly rely on legal protection against dismissal during pregnancy and parental leave, which reduces the objective risk of job loss during this period. In addition, the labor market for well-trained workers in Germany is comparatively good, which could make a potential job loss seem less threatening due to the good chances of finding a new job. Therefore, job insecurity may have less weight in our specific sample of highly educated German workers in the transition to parenthood. As we entered abusive supervision, job insecurity, and job demand simultaneously in one model, the limited effect could also be explained by the overlapping variance of job insecurity with abusive supervision, supported by their significant moderate correlation.

Results for the predictive value of *job demand* were also mixed. While job demand significantly predicted symptoms of somatization in mothers, even after controlling for pre-existing symptoms of somatization during pregnancy, the significant predictive association with symptoms of anxiety was no longer significant after controlling for pre-existing symptoms of anxiety. Furthermore, job demand did not significantly predict any of the other mental health outcomes in mothers, and none of the mental health outcomes in partners. Moreover, only the predictive relationship with symptoms of somatization in mothers *before* controlling for prior symptoms remained significant after Šidák correction. While these findings are in line with a study in which job demand was no relevant predictor after controlling for other variables [78, 135], they contradict former literature identifying job demand as an important predictor of parental mental health in the transition to parenthood [81] and the general population [6]. A possible explanation for these mixed findings is provided by the challenge-hindrance framework [136, 137], which suggests that job demands must be distinguished into challenge stressors (i.e., appraised as potentially promoting achievement) and hindrance stressors (i.e., appraised as potentially threatening achievement). Research indicates that challenge appraisals are positively associated with favorable health outcomes, whereas hindrance appraisals are negatively associated with favorable health outcomes [138]. However, the appraisal process of job demands is not straightforward. Gerich and Weber [136] report that it is moderated by perceived job control and social support at work and that job demands can simultaneously incorporate challenge and hindrance components. Furthermore, the relation between demand intensity and challenge appraisal is reported to be curvilinear, suggesting that too low job demands can even deteriorate health outcomes via feelings of frustration or boredom [136, 137]. As we did not assess individual challenge/hindrance appraisals or perceptions of control and social support at work, we could not account for their potential influence. Given the relatively high level of education in our sample, many parents may hold jobs that offer a high degree of control [139]. This perceived job control may facilitate challenge appraisals, which could explain the lack of significant associations between job demand and mental health outcomes. Overall, our results on job demand are in line with the conflicting results of previous studies and the possibility that its influence depends on other mediating/moderating variables, such as job resources (e.g., supervisor support, and job control; [135]).

As reported above, many prospective associations of working conditions remained significant even when *controlling for pre-existing symptomatology* (i.e., precarious employment as a predictor for mothers’ and partners’ symptoms of obsessive-compulsiveness and anxiety, and mothers’ symptoms of somatization and anger/hostility; abusive supervision as a predictor for all mental health outcomes in mothers and partners; and job demand as a predictor for mothers’ symptoms of somatization). After Šidák correction, this held for: (1) precarious employment predicting mothers’ symptoms of somatization, obsessive-compulsiveness, anxiety, and anger/hostility; and (2) abusive supervision predicting symptoms of depression, obsessive-compulsiveness, and anxiety in both mothers and partners, as well as partners’ symptoms of anger/hostility. This demonstrates so-called “Granger causality” [140], meaning that working conditions have a predictive effect on mental health over time *beyond the initial symptom level*. Importantly, that some of the relationships were no longer significant when adjusting for pre-existing symptoms does not imply that the working condition is irrelevant. Potential influences could have already manifested at T1 or earlier and thus do not lead to further deterioration.

Our multiple regression analyses showed *similar patterns of associations for the different mental health symptoms* considered, indicating that adverse working conditions might have an impact on mental health *in general* and contribute to a *general vulnerability* [53, 76]. The fact that adverse working conditions also predicted symptoms of somatization, obsessive-compulsiveness, and anger/hostility – which have received much less attention than symptoms of depression or anxiety in the literature – emphasizes the importance of considering a range of symptoms in research and practice so that no affected parents are overlooked. In addition, our results evidence *persistent and/or delayed effects* (i.e., adverse working conditions measured *during pregnancy* prospectively predicted mental health *many months later*). Together, these findings align with the COR Theory [64, 65], which proposes that adverse prepartum working conditions reduce the parents’ resources to cope with upcoming strains and thereby might hinder a successful adjustment in the long term. The consideration that adverse working conditions might unfold their impact on mental health symptoms as a general stressor consuming available coping resources is particularly worrying as the transition to parenthood is already a vulnerable period in terms of mental health with many challenges, and (expectant) parents may therefore be particularly affected by adverse working conditions. For a better understanding of the hereby suggested stress-related mechanisms underlying the influence of adverse working conditions on parental mental health in the transition to parenthood, it is imperative to include stress biomarkers in research. For instance, Julià et al. [56] reported that precarious employment is related to employees’ stress biomarkers of adrenal and gonadal hormone production, including cortisol. So far, research on new parents within this context is lacking.

Research on peripartum mental health in partners is generally scarce. However, this gap might be particularly relevant in the context of work factors, as the work environment usually remains present for partners throughout the transition to parenthood, whereas mothers typically plan for longer periods of parental leave after childbirth. Parental leave may buffer the effects of adverse prepartum working conditions, as they become more distant from the postpartum period. The findings of a recent study indeed support that prepartum work factors might be of greater relevance for paternal than maternal postpartum mental health symptoms [57]. However, in the current study, *working conditions predicted mental health symptoms in a similar way in both mothers and partners*. Minor differences pertain to precarious employment, job insecurity, and job demand predicting slightly more mental health symptoms in mothers than in partners (98.5% males). This contradictory finding might be explained by differential exposure and/or gender inequalities concerning the perception and appraisal of working characteristics – for instance, women are more likely than men to be employed precariously or have jobs with less prestige and control [53, 141]. In line with the considerations above, whether job demands are rather appraised as a hindrance or challenge might differ between mothers and partners due to differential exposure to jobs with low control or autonomy, which has been found to moderate or mediate the association between adverse working conditions and mental health symptoms [136, 142] as well as gender and depressive symptoms [143]. Overall, the results underscore that we need to take both mothers and their partners into focus when examining work factors influencing peripartum mental health. This need is reinforced by the fact that dyadic effects are to be expected, as partners’ mental health was found to be interdependent [144], meaning that poor working conditions of one partner could indirectly affect the mental health of the other partner. Further dyadic research is needed to study these potential dyadic effects related to working conditions.

### Strengths and limitations

This study is the first to examine precarious employment and abusive supervision as prospective risk factors for mothers’ *and* partners’ mental health in the transition to parenthood. Further strengths include our large sample size, the longitudinal approach, the use of validated measures for work and mental health variables, and the multiple theoretical approaches in assessing working conditions. In addition, we considered a wide range of potential mental health symptoms, including symptoms of depression and anxiety, but also less-often-studied symptoms of somatization, obsessive-compulsiveness, and anger/hostility. Moreover, in the second step of our hierarchical multiple regression analyses, we controlled for pre-existing symptoms of the respective mental health variable during pregnancy, and many of the effects remained significant in these models. Thus, we establish “Granger causality” [140], indicating that working conditions during pregnancy provide information that helps predict mental health outcomes 14 months postpartum, beyond the predictive power of mental health status during pregnancy alone. Another strength is that we considered COVID-19 pandemic exposure as a potential confounder due to the reported pandemic-related burden on peripartum mental health (e.g., [125, 145]). As there were no significant correlations with our outcome measures, COVID-19 pandemic exposure was not included in our main analyses. Finally, our rigorous approach to multivariate outliers (i.e., detecting multivariate outliers using Mahalanobis distance, conducting sensitivity analyses with outliers included to examine their influence, and deciding to exclude them from the main analyses) ensures that the results of the regression analyses, which are not robust to outliers, are not distorted by their influence. As the sensitivity analyses revealed minor differences, particularly in Model 2 and in the direction of additional significant associations, we are confident that our approach provides the most robust estimates for the prospective associations investigated.

Notwithstanding, the current study has some limitations. Firstly, some findings did not remain statistically significant after the Šidák correction and should therefore be interpreted with caution. However, the correction did not alter the overall pattern of predictive relationships, supporting the robustness of our conclusions while addressing multiple testing concerns. Furthermore, changes in the working situation after their assessment during pregnancy were not included in our analyses because a substantial portion of our sample, particularly mothers, had not (yet) returned to work at the time of the postpartum survey (*n* not working: mothers = 556, 44.2%; partners = 76, 9.4%). Additionally, we did not assess whether participants remained in the same employment contract, which would have been necessary to accurately capture changes in the working conditions over time. Consequently, some participants may have experienced better or worse working conditions or were not working at the time of their mental health assessment. Nevertheless, the significant prospective associations we found emphasize the long-term centrality of working conditions during pregnancy for postpartum mental health, regardless of current working conditions. Moreover, due to the predominantly high educational level of the sample, our results are especially generalizable to a more highly educated population. It can be assumed that education is intertwined with the investigated relationships due to interrelations with occupational position and exposure to precarity in work [146]. Our finding that working conditions play an important role in the adjustment to parenthood, even among highly educated (expectant) parents less exposed to precarious conditions, is nonetheless important and concerns many families and – through the workers’ contribution to the economy – also society as a whole [147]. Attrition analyses revealed that participants with no academic degree, poorer mental health, and higher scores of precarious employment and abusive supervision measured during pregnancy were more likely to drop out of the study, suggesting attrition-related "missing at random" (MAR). While we cannot entirely rule out "missing not at random" (MNAR), as distinguishing MAR from MNAR is inherently impossible, we have no evidence for MNAR and consider it rather unlikely. Nevertheless, our results may even underestimate the true relationships between precarious employment and abusive supervision and mental health. These findings indicate that it is crucial to identify expectant parents who are experiencing particularly adverse working conditions or existing mental health problems at an early stage, in order to prevent them from being overlooked and to ensure that they receive the necessary support. Regarding missing data handling within the final sample, we applied person mean imputation for participants missing up to 20% of items on a scale. While we acknowledge that methods such as multiple imputation or full information maximum likelihood are generally more robust techniques, person mean imputation was deemed appropriate in our case due to the very low overall rates of missing data (< 1%, see Table S3 in Additional file 1), which makes potential bias likely negligible, coupled with its greater practicability.

The use of subjective ratings of working conditions implies that ratings depend on individual expectations and standards (e.g., what authoritarian behavior by supervisors entails or what an increasing workload means personally). However, we did use the recommended multidimensional measure of precarious employment including objective data such as type of contract, permanent vs. temporary employment, or salary [53]. Furthermore, research indicates that both objective aspects as well as perceived dimensions of working conditions can impact workers’ mental health (e.g., [142, 148]), suggesting that a combined approach, as in our study, is indeed most suitable.

### Implications

The current study underscores the importance of considering adverse working conditions as significant risk factors for mental health problems during the transition to parenthood. Policymakers should prioritize reducing the prevalence of mental health problems in new parents by implementing stronger protections against adverse working conditions – specifically precarious employment and abusive supervision – during the transition to parenthood. This could prevent adverse outcomes for both parents [149] and, in turn, also children [45, 90, 107]. Moreover, promoting strategies against precarious employment could help reduce health inequalities [143, 150].

Clinicians should consider the findings of this study in prenatal care by inquiring about the working conditions and social environment at work for pregnant women and their partners. Parents who are already vulnerable before the birth of their child may be especially susceptible to adverse working conditions, as their resources to cope with challenges are more limited [86, 151]. In contrast, parents with abundant resources may better manage these additional stressors. Identifying individuals at risk allows for targeted preventive interventions, such as psychological counseling on stress management. Developing screening methods for clinical settings to identify these at-risk individuals is crucial for effective early intervention.

In future research, it would be interesting to explore cross-country comparisons, as government regulations (e.g., regarding parental leave policies) could modulate the effects of adverse working conditions. Additionally, further studies could enhance a more differentiated understanding by comparing the mental health impact of working conditions among new parents, parents with older children, and individuals without children. Examining longitudinal cross-lagged changes in both working conditions and mental health, especially around the birth of a child, would provide valuable insights regarding potential causal directions. Furthermore, studies incorporating biomarkers of stress responses will be beneficial in elucidating the physiological mechanisms underlying the impact of working conditions on parental mental health. Such investigations are part of the DREAM sub-studies DREAM_HAIR_ and DREAM_EPI_, with the latter also focusing on the stress-related effects on offspring [for further information see study protocol; 108], and related publications currently under review. In addition, further research explicitly examining dyadic couple data, as in the study by Kress et al. [57], will provide a more thorough understanding of how working conditions impact *family* mental health by considering the interconnected experiences of both partners during the transition to parenthood [152]. Finally, future research could contribute to a deeper understanding of the role of working conditions for (expectant) parents’ mental health by integrating a more comprehensive range of established work-related *mediating/moderating* protective and risk factors into a model that incorporates our findings and testing hypotheses about potential mechanisms. Among these factors, work-family/family-work conflict, relationships with co-workers, perceptions of control, autonomy, flexibility, and social support [83, 84, 87] could be especially instructive.

Mental health problems in new parents can have profound implications, not only for the affected individuals but also for the healthy development of their offspring [45, 90, 107], as well as for society as a whole, due to the high costs associated with mental health issues [9–13]. Therefore, addressing the work-related factors contributing to these problems is not only critical for individual families but also for broader societal well-being and public mental health.

## Conclusions

Our findings provide new evidence that adverse working conditions can act as risk factors for first-time parents’ mental health during the vulnerable transition to parenthood. In particular, precarious employment and abusive supervision before or during pregnancy emerged as important predictors of mental health symptoms 14 months postpartum. These results underscore the urgency of further research into the underlying mechanisms affecting both mothers and their partners. Work policies promoting beneficial working conditions are imperative. Additionally, our study highlights the critical need for targeted preventive measures during pregnancy to mitigate potential adverse effects. Addressing the identified work-related risk factors is essential for promoting the well-being of new parents and their families.

## Supplementary Information

Additioanl file 1.

Additional file 2.

Additional file 3.

Additional file 4.

## Data Availability

The dataset presented in this article is not publicly available because of legal and ethical constraints. Public sharing of participant data was not included in the informed consent of the study. Requests to access the datasets should be directed to the project manager and principal investigator Susan Garthus-Niegel.

## Abbreviations

DREAM: DResdner Studie zu Elternschaft, Arbeit und Mentaler Gesundheit; Dresden Study on Parenting, Work, and Mental Health
OECD: Organisation for Economic Co-operation and Development
COR: Conservation of Resources
COVID-19: Coronavirus Disease 2019
EPRES: Employment Precariousness Scale
ERI: Effort-Reward Imbalance Questionnaire
EPDS: Edinburgh Postnatal Depression Scale
SCL-90-R: Symptom-Checklist Revised
MAR: Missing at random
MNAR: Missing not at random
