# Supplementary material for: Prepartum working conditions predict mental health symptoms 14 months postpartum in first-time mothers and their partners – results of the prospective cohort study “DREAM”
Source: BMC Public Health. 2025 Mar 5;25:875. doi: 10.1186/s12889-025-21886-2 (PMC11884048; doi:10.1186/s12889-025-21886-2)
Supplement: Supplementary file 1 — Additioanl file 1. [file 12889_2025_21886_MOESM1_ESM.docx]

# Additional file 1. Attrition analyses and rates of missing data in the final sample

**Table S1** Results of attrition analyses with *t*-tests

| **Variable** |  | **Mothers** | | | | |  | **Partners** | | | | |
| --- | --- | --- | --- | --- | --- | --- | --- | --- | --- | --- | --- | --- |
|  |  | *n ^a^* | *M* ± *SD* | *t*(df) | *p* | Cohen’s *d* |  | *n ^a^* | *M* ± *SD* | *t*(df) | *p* | Cohen’s *d* |
| **Age** (years) | Completer  Non-Completer | 1278  266 | 29.9 ± 3.62  29.8 ± 4.00 | −0.15(361) | .881 | −0.01 |  | 843  247 | 32.1 ± 4.43  31.5 ± 4.08 | −1.80(1088) | .058 | −1.30 |
| **Precarious employment** (EPRES) | Completer  Non-Completer | 1191  237 | 0.96 ± 0.45  1.02 ± 0.42 | 2.20(1426) | **.020** | 0.16 |  | 751  210 | 0.77 ± 0.40  0.88 ± 0.46 | 3.25(959) | **.003** | 0.25 |
| **Abusive supervision** (EPRES subscale) | Completer  Non-Completer | 1272  262 | 0.78 ± 0.72  0.83 ± 0.69 | .93(1532) | .327 | 0.06 |  | 832  249 | 0.54 ± 0.59  0.64 ± 0.65 | 2.24(1079) | **.032** | 0.16 |
| **Job insecurity**  (ERI subscale) | Completer  Non-Completer | 1273  261 | 3.73 ± 1.37  3.67 ± 1.31 | −0.69(1532) | .487 | −0.05 |  | 838  249 | 3.31 ± 1.22  3.36 ± 1.22 | 0.53(1085) | .593 | 0.04 |
| **Job demand**  (ERI subscale) | Completer  Non-Completer | 1269  258 | 5.87 ± 1.38  5.85 ± 1.35 | −0.14(1525) | .883 | −0.01 |  | 842  249 | 5.77 ± 1.29  5.72 ± 1.29 | −0.51(1089) | .603 | −0.04 |
| **Symptoms of** |  |  |  |  |  |  |  |  |  |  |  |  |
| **Depression** (EPDS) | Completer  Non-Completer | 1284  266 | 5.31 ± 3.86  6.06 ± 4.31 | 2.85(1548) | **.007** | 0.19 |  | 845  249 | 3.49 ± 3.29  4.27 ± 3.29 | 2.95 (367) | **.003** | 0.23 |
| **Somatization**  (SCL-90-R subscale) | Completer  Non-Completer | 1279  267 | 5.58 ± 4.63  6.05 ± 4.63 | 1.48(1544) | .132 | 0.10 |  | 839  248 | 2.22 ± 2.61  2.51 ± 2.69 | 1.52 (1085) | .128 | 0.11 |
| **Obsessive-compulsiveness**  (SCL-90-R subscale) | Completer  Non-Completer | 1283  267 | 4.36 ± 4.00  5.27 ± 4.55 | 3.03(357) | **.003** | 0.22 |  | 840  249 | 2.96 ± 3.63  3.36 ± 3.55 | 1.56 (1087) | .114 | 0.11 |
| **Anxiety**  (SCL-90-R subscale) | Completer  Non-Completer | 1278  267 | 2.54 ± 3.01  3.05 ± 3.97 | 1.96(333) | .057 | 0.16 |  | 839  248 | 1.56 ± 2.17  1.87 ± 2.39 | 1.83(375) | .069 | 0.14 |
| **Anger/hostility**  (SCL-90-R subscale) | Completer  Non-Completer | 1279  267 | 1.96 ± 2.14  2.43 ± 2.57 | 2.77(347) | **.007** | 0.21 |  | 838  248 | 1.22 ± 1.89  1.55 ± 2.03 | 2.27(383) | **.029** | 0.17 |

*Note*. Variables measured at T1 (during pregnancy). EPRES = Employment Precariousness Scale, ERI = Effort-Reward Imbalance Questionnaire, EPDS = Edinburgh Postnatal Depression Scale, SCL-90-R = Symptom Checklist-90-Revised.
^a^ *n* varies slightly due to missing data of some participants.
*p*-values based on 5,000 bootstrap samples; *p*-values < .05 are presented in bold.

**Table S2** Results of attrition analyses with Chi-square tests

| **Variable** |  | **Mothers** | | | |  | **Partners** | | | |
| --- | --- | --- | --- | --- | --- | --- | --- | --- | --- | --- |
|  |  | *n ^a^* | df | χ^2^ | *p* |  | *n ^a^* | df | χ^2^ | *p* |
| **Academic degree ^b^** | Completer  Non-Completer | 1283  265 | 1 | 5.93 | **.015** |  | 839  249 | 1 | 33.43 | **<.001** |
| **Country of birth ^c^** | Completer  Non-Completer | 1284  266 | 1 | 4.61 | **.032** |  | 845  251 | 1 | 9.99 | **.002** |
| **Marital status** | Completer  Non-Completer | 1281  265 | 4 | 2.86 ^d^ | .581 |  | 848  250 | 3 | 7.52 ^d^ | .064 |
| **Employment status before pregnancy** | Completer  Non-Completer | 1288  267 | 2 | 2.00 | .367 |  | 849  252 | 2 | 0.06 | .968 |
| **Employment status during pregnancy** | Completer  Non-Completer | 1288  267 | 3 | 1.03 | .794 |  | 849  252 | 2 | 0.18 | .915 |

*Note*. Variables measured at T1 (during pregnancy).
^a^ *n* varies slightly due to missing data of some participants. ^b^ Non-completers had significantly less often an academic degree than completers. ^c^ Non-completers were born significantly more often in another country than Germany than completers. ^d^ More than 20% of the cells have an expected frequency of less than 5.
*p*-values < .05 are presented in bold.

**Table S3** Rates of missing data in the final sample (*N* = 2,070)

| **Variable** | Range of missing data per item within associated scale (%) | Total percentage of missing data per scale | Participants with ≤ 20% missing data per scale, *n* (%) | Participants with > 20% missing data per scale, *n* (%) |
| --- | --- | --- | --- | --- |
| **Precarious employment** (EPRES) | 0.1–3.2 | 0.73 | 165 (8.0) | 6 (0.3) |
| **Abusive supervision** (EPRES subscale) | 0.3–0.5 | 0.44 | 21 (1.0) | 7 (0.3) |
| **Job insecurity**  (ERI subscale) | 0.6–0.8 | 0.68 | - | 22 (1.1) |
| **Job demand**  (ERI subscale) | 0.4–0.9 | 0.65 | - | 24 (1.2) |
| **Symptoms of** |  |  |  |  |
| **Depression** (EPDS) | 0.1–0.4 | 0.25 | 28 (1.4) | 3 (0.1) |
| **Somatization** (SCL-90-R subscale) | 0.6–0.9 | 0.76 | 50 (2.4) | 13 (0.6) |
| **Obsessive-compulsiveness** (SCL-90-R subscale) | 0.8–1.2 | 0.88 | 59 (2.9) | 13 (0.6) |
| **Anxiety** (SCL-90-R subscale) | 0.6–0.9 | 0.74 | 30 (1.4) | 14 (0.7) |
| **Anger/hostility** (SCL-90-R subscale) | 0.7–1.1 | 0.81 | 29 (1.4) | 14 (0.7) |

*Note*. EPRES = Employment Precariousness Scale, ERI = Effort-Reward Imbalance Questionnaire, EPDS = Edinburgh Postnatal Depression Scale, SCL-90-R = Symptom Checklist-90-Revised.
