# Supplementary material for: Prepartum working conditions predict mental health symptoms 14 months postpartum in first-time mothers and their partners – results of the prospective cohort study “DREAM”
Source: BMC Public Health. 2025 Mar 5;25:875. doi: 10.1186/s12889-025-21886-2 (PMC11884048; doi:10.1186/s12889-025-21886-2)
Supplement: Supplementary file 2 — Additional file 2. [file 12889_2025_21886_MOESM2_ESM.docx]

# Additional file 2. Preliminary correlation analyses of potential confounding variables

**Table S1** Preliminary correlation analyses of potential confounding variables with outcomes (prior to exclusion of multivariate outliers)

| **Variable** | **Mothers’ symptoms of** | | | | |  | **Partners’ symptoms of** | | | | |
| --- | --- | --- | --- | --- | --- | --- | --- | --- | --- | --- | --- |
|  | **DEP** | **SOM** | **OC** | **ANX** | **A/H** |  | **DEP** | **SOM** | **OC** | **ANX** | **A/H** |
| **Age (years)** | −.07^*^ | −.06^*^ | −.05 | −.04 | −.06^*^ |  | .00 | .02 | .01 | .04 | .00 |
| **Employment status T1^a^** | .07^*^ | .05 | .05 | .06^*^ | .05 |  | .12^**^ | .05 | .09^*^ | .03 | .02 |
| **Duration of parental leave up to T3 (months)** | −.05 | −.04 | −.06^*^ | −.09^**^ | −.06^*^ |  | .00 | .00 | −.01 | −.05 | −.01 |
| **Academic degree ^b^** | −.04 | −.06^*^ | −.01 | −.02 | −.02 |  | .01 | −.09^*^ | .04 | .01 | −.03 |
| **T3 Covid-19 pandemic exposure ^c^** | .01 | −.02 | .01 | −.02 | .04 |  | .02 | .00 | −.01 | −.00 | −.05 |

*Note*. DEP = Depression, SOM = Somatization, OC = Obsessive-Compulsiveness, ANX = Anxiety, A/H = Anger/Hostility.
 ^a^ 0 = full-time and 1 = part-time or marginal; If mothers were in employment ban at T1, employment status before employment ban was used. ^b^ 0 = no academic degree and 1 = academic degree. ^c^ Two categories based on the date of completion at T3, i.e., when the outcome variable was assessed. Those who completed T3 between March 10th, 2020, and January 15th, 2023 were assigned to the “during pandemic” group = 1, otherwise, they were placed in the “before/after pandemic” group = 0.
^*^*p* < .05, ^**^*p* < .01.
