# Supplementary material for: Prepartum working conditions predict mental health symptoms 14 months postpartum in first-time mothers and their partners – results of the prospective cohort study “DREAM”
Source: BMC Public Health. 2025 Mar 5;25:875. doi: 10.1186/s12889-025-21886-2 (PMC11884048; doi:10.1186/s12889-025-21886-2)
Supplement: Supplementary file 3 — Additional file 3. [file 12889_2025_21886_MOESM3_ESM.docx]

# Additional file 3. Regression analyses including confounding variables

**Table S1** Hierarchical multiple regression analyses predicting T3 symptoms of depression from T1 working conditions and baseline symptoms of depression

|  | **Mothers** | | | | **Partners** | | | |
| --- | --- | --- | --- | --- | --- | --- | --- | --- |
|  | Model 1a | Model 2a | Model 1b | Model 2b | Model 1a | Model 2a | Model 1b | Model 2b |
| Age | −.04 | −.04 | −.06^*^ | −.04 | x | x | x | x |
| Duration of parental leave up to T3 (months) | .00 | .00 | −.03 | −.00 | x | x | x | x |
| Academic degree ^a^ | .02 | .06^*^ | .02 | .05 | .00 | −.01 | .03 | .01 |
| Employment status T1 ^b^ | .01 | .01 | .05 | .02 | .07 | .08 | .12^**^ | .11^**^ |
| Symptoms of depression T1 (EPDS) | x | .49^**^ | x | .48^**^ | x | .49^**^ | x | .48^**^ |
| Precarious employment (EPRES) | .14^**^ | .05 | x | x | .10^*^ | .04 | x | x |
| Abusive supervision (EPRES subscale) | x | x | .18^**^ | .09^**^ | x | x | .24^**^ | .12^**^ |
| Job insecurity (ERI subscale) | x | x | .10^**^ | .04 | x | x | .10^*^ | .02 |
| Job demand (ERI subscale) | x | x | .02 | −.01 | x | x | .00 | −.03 |
| *R*² (adjusted) | .02 | .25 | .06 | .27 | .01 | .25 | .09 | .29 |

*Note*. Standardized βs are reported. Hierarchical multiple regression analyses with working condition(s) and sociodemographic confounders age (only mothers), duration of parental leave (only mothers), educational level, and employment status entered in a first step (Model 1) and baseline (T1) symptoms of respective mental health outcomes added in the second step (Model 2). In Model 1a and 2a, precarious employment was entered as working condition; in Model 1b and 2b, abusive supervision, job insecurity, and job demand were entered simultaneously as working conditions. Symptoms of depression were measured with the Edinburgh Postnatal Depression Scale (EPDS). T1 = during pregnancy, T3 = 14 months postpartum. EPRES = Employment Precariousness Scale, ERI = Effort-Reward Imbalance Questionnaire.

^a^ 0 = no academic degree and 1 = academic degree. ^b^ 0 = full-time, 1 = part-time or marginal; if mothers were in employment ban at T1, employment status before employment ban was used.

^*^*p* < .05; ^**^*p* < .01; *p*-values based on 5,000 bootstrap samples.

**Table S2** Hierarchical multiple regression analyses predicting T3 symptoms of somatization from T1 working conditions and baseline symptoms of somatization

|  | Mothers | | | | Partners | | | |
| --- | --- | --- | --- | --- | --- | --- | --- | --- |
|  | Model 1a | Model 2a | Model 1b | Model 2b | Model 1a | Model 2a | Model 1b | Model 2b |
| Age | −.01 | .02 | −.04 | .01 | x | x | x | x |
| Duration of parental leave up to T3 (months) | −.03 | −.03 | −.03 | −.03 | x | x | x | x |
| Academic degree ^a^ | −.02 | .01 | −.01 | .01 | −.05 | −.03 | −.05 | −.03 |
| Employment status T1 ^b^ | −.01 | .01 | .02 | .02 | −.01 | −.02 | .04 | .02 |
| Symptoms of somatization T1 (SCL-90-R) | x | .44^**^ | x | .42^**^ | x | .47^**^ | x | .49^**^ |
| Precarious employment (EPRES) | .15^**^ | .09^**^ | x | x | .12^*^ | .08 | x | x |
| Abusive supervision (EPRES subscale) | x | x | .14^**^ | .07^*^ | x | x | .20^**^ | .09^*^ |
| Job insecurity (ERI subscale) | x | x | .08^*^ | .03 | x | x | .00 | .00 |
| Job demand (ERI subscale) | x | x | .09^**^ | .07^*^ | x | x | .02 | −.01 |
| *R*² (adjusted) | .02 | .20 | .05 | .21 | .01 | .23 | .04 | .26 |

*Note*. Standardized βs are reported. Hierarchical multiple regression analyses with working condition(s) and sociodemographic confounders age (only mothers), duration of parental leave (only mothers), educational level, and employment status entered in a first step (Model 1) and baseline (T1) symptoms of respective mental health outcomes added in the second step (Model 2). In Model 1a and 2a, precarious employment was entered as working condition; in Model 1b and 2b, abusive supervision, job insecurity, and job demand were entered simultaneously as working conditions. Symptoms of somatization were measured with the corresponding subscale of the Symptom Checklist-90-Revised (SCL-90-R). T1 = during pregnancy, T3 = 14 months postpartum. EPRES = Employment Precariousness Scale, ERI = Effort-Reward Imbalance Questionnaire.

^a^ 0 = no academic degree and 1 = academic degree. ^b^ 0 = full-time, 1 = part-time or marginal; if mothers were in employment ban at T1, employment status before employment ban was used.

^*^*p* < .05; ^**^*p* < .01; *p*-values based on 5,000 bootstrap samples.

**Table S3** Hierarchical multiple regression analyses predicting T3 symptoms of obsessive-compulsiveness (OC) from T1 working conditions and baseline symptoms of obsessive-compulsiveness

|  | Mothers | | | | Partners | | | |
| --- | --- | --- | --- | --- | --- | --- | --- | --- |
|  | Model 1a | Model 2a | Model 1b | Model 2b | Model 1a | Model 2a | Model 1b | Model 2b |
| Age | −.01 | .01 | −.04 | .00 | x | x | x | x |
| Duration of parental leave up to T3 (months) | −.01 | −.04 | −.03 | −.04 | x | x | x | x |
| Academic degree ^a^ | .04 | .05 | .04 | .04 | .06 | .03 | .08^*^ | .04 |
| Employment status T1 ^b^ | .00 | .01 | .03 | .02 | −.02 | −.03 | .06 | .02 |
| Symptoms of OC T1 (SCL-90-R) | x | .50^**^ | x | .47^**^ | x | .52^**^ | x | .55^**^ |
| Precarious employment (EPRES) | .18^**^ | .08^**^ | x | x | .15^**^ | .07^*^ | x | x |
| Abusive supervision (EPRES subscale) | x | x | .20^**^ | .09^*^ | x | x | .28^**^ | .11^**^ |
| Job insecurity (ERI subscale) | x | x | .07^*^ | .03 | x | x | .01 | −.04 |
| Job demand (ERI subscale) | x | x | .02 | .00 | x | x | −.05 | −.03 |
| *R*² (adjusted) | .03 | .26 | .05 | .25 | .02 | .28 | .07 | .34 |

*Note*. Standardized βs are reported. Hierarchical multiple regression analyses with working condition(s) and sociodemographic confounders age (only mothers), duration of parental leave (only mothers), educational level, and employment status entered in a first step (Model 1) and baseline (T1) symptoms of respective mental health outcomes added in the second step (Model 2). In Model 1a and 2a, precarious employment was entered as working condition; in Model 1b and 2b, abusive supervision, job insecurity, and job demand were entered simultaneously as working conditions. Symptoms of OC were measured with the corresponding subscale of the Symptom Checklist-90-Revised (SCL-90-R). T1 = during pregnancy, T3 = 14 months postpartum. EPRES = Employment Precariousness Scale, ERI = Effort-Reward Imbalance Questionnaire.

^a^ 0 = no academic degree and 1 = academic degree. ^b^ 0 = full-time, 1 = part-time or marginal; if mothers were in employment ban at T1, employment status before employment ban was used.

^*^*p* < .05; ^**^*p* < .01; *p*-values based on 5,000 bootstrap samples.

**Table S4** Hierarchical multiple regression analyses predicting T3 symptoms of anxiety from T1 working conditions and baseline symptoms of anxiety

|  | Mothers | | | | Partners | | | |
| --- | --- | --- | --- | --- | --- | --- | --- | --- |
|  | Model 1a | Model 2a | Model 1b | Model 2b | Model 1a | Model 2a | Model 1b | Model 2b |
| Age | −.04 | −.04 | −.05 | −.04 | x | x | x | x |
| Duration of parental leave up to T3 (months) | −.03 | −.05 | −.05 | −.05^*^ | x | x | x | x |
| Academic degree ^a^ | .04 | .05 | .05 | .04 | .04 | .01 | .04 | .00 |
| Employment status T1 ^b^ | .05 | .06 | .09^**^ | .09^**^ | −.03 | −.03 | .03 | .01 |
| Symptoms of anxiety T1 (SCL-90-R) | x | .40^**^ | x | .38^**^ | x | .46^**^ | x | .49^**^ |
| Precarious employment (EPRES) | .14^**^ | .09^**^ | x | x | .12^**^ | .07^*^ | x | x |
| Abusive supervision (EPRES subscale) | x | x | .19^**^ | .10^**^ | x | x | .27^**^ | .14^**^ |
| Job insecurity (ERI subscale) | x | x | .05 | .03 | x | x | .02 | −.02 |
| Job demand (ERI subscale) | x | x | .07^*^ | .04 | x | x | −.03 | −.06 |
| *R*² (adjusted) | .03 | .18 | .06 | .19 | .01 | .22 | .07 | .28 |

*Note*. Standardized βs are reported. Hierarchical multiple regression analyses with working condition(s) and sociodemographic confounders age (only mothers), duration of parental leave (only mothers), educational level, and employment status entered in a first step (Model 1) and baseline (T1) symptoms of respective mental health outcomes added in the second step (Model 2). In Model 1a and 2a, precarious employment was entered as working condition; in Model 1b and 2b, abusive supervision, job insecurity, and job demand were entered simultaneously as working conditions. Symptoms of anxiety were measured with the corresponding subscale of the Symptom Checklist-90-Revised (SCL-90-R). T1 = during pregnancy, T3 = 14 months postpartum. EPRES = Employment Precariousness Scale, ERI = Effort-Reward Imbalance Questionnaire.

^a^ 0 = no academic degree and 1 = academic degree. ^b^ 0 = full-time, 1 = part-time or marginal; if mothers were in employment ban at T1, employment status before employment ban was used.

^*^*p* < .05; ^**^*p* < .01; *p*-values based on 5,000 bootstrap samples.

**Table S5** Hierarchical multiple regression analyses predicting T3 symptoms of anger/hostility from T1 working conditions and baseline symptoms of anger/hostility

|  | Mothers | | | | Partners | | | |
| --- | --- | --- | --- | --- | --- | --- | --- | --- |
|  | Model 1a | Model 2a | Model 1b | Model 2b | Model 1a | Model 2a | Model 1b | Model 2b |
| Age | −.06^*^ | −.02 | −.07^**^ | −.03 | x | x | x | x |
| Duration of parental leave up to T3 (months) | −.04 | −.04 | −.05 | −.04 | x | x | x | x |
| Academic degree ^a^ | .02 | .03 | .03 | .03 | −.03 | −.02 | .00 | .00 |
| Employment status T1 ^b^ | .00 | .00 | .02 | .01 | −.04 | −.02 | .04 | .03 |
| Symptoms of anger/hostility T1 (SCL-90-R) | x | .42^**^ | x | .43^**^ | x | .47^**^ | x | .43^**^ |
| Precarious employment (EPRES) | .14^**^ | .09^**^ | x | x | .09^*^ | .04 | x | x |
| Abusive supervision (EPRES subscale) | x | x | .17^**^ | .08^*^ | x | x | .24^**^ | .12^**^ |
| Job insecurity (ERI subscale) | x | x | .04 | .03 | x | x | −.01 | −.04 |
| Job demand (ERI subscale) | x | x | .02 | .02 | x | x | −.01 | −.04 |
| *R*² (adjusted) | .02 | .19 | .04 | .21 | .00 | .22 | .05 | .22 |

*Note*. Standardized βs are reported. Hierarchical multiple regression analyses with working condition(s) and sociodemographic confounders age (only mothers), duration of parental leave (only mothers), educational level, and employment status entered in a first step (Model 1) and baseline (T1) symptoms of respective mental health outcomes added in the second step (Model 2). In Model 1a and 2a, precarious employment was entered as working condition; in Model 1b and 2b, abusive supervision, job insecurity, and job demand were entered simultaneously as working conditions. Symptoms of anger/hostility were measured with the corresponding subscale of the Symptom Checklist-90-Revised (SCL-90-R). T1 = during pregnancy, T3 = 14 months postpartum. EPRES = Employment Precariousness Scale, ERI = Effort-Reward Imbalance Questionnaire.

^a^ 0 = no academic degree and 1 = academic degree. ^b^ 0 = full-time, 1 = part-time or marginal; if mothers were in employment ban at T1, employment status before employment ban was used.

^*^*p* < .05; ^**^*p* < .01; *p*-values based on 5,000 bootstrap samples.
