# Supplementary material for: Prepartum working conditions predict mental health symptoms 14 months postpartum in first-time mothers and their partners – results of the prospective cohort study “DREAM”
Source: BMC Public Health. 2025 Mar 5;25:875. doi: 10.1186/s12889-025-21886-2 (PMC11884048; doi:10.1186/s12889-025-21886-2)
Supplement: Supplementary file 4 — Additional file 4. [file 12889_2025_21886_MOESM4_ESM.docx]

# Additional file 4. Sensitivity analyses – Correlation matrix and regression analyses with outliers included

**Table S1** Correlation matrix of study variables of mothers (above diagonal) and partners (below diagonal) with multivariate outliers included

| **Variable** | | **1** | **2** | **3** | **4** | **5** | **6** | **7** | **8** | **9** | **10** | **11** | **12** | **13** | **14** | **15** | **16** | **17** | **18** |
| --- | --- | --- | --- | --- | --- | --- | --- | --- | --- | --- | --- | --- | --- | --- | --- | --- | --- | --- | --- |
| 1) | **Precarious employment T1** (EPRES) | − | .58^**^ | .36^**^ | .09^**^ | .20^**^ | .21^**^ | .22^**^ | .22^**^ | .19^**^ | −.25^**^ | −.10^**^ | −.18^**^ | .23^**^ | .24^**^ | .20^**^ | .23^**^ | .17^**^ | .17^**^ |
| 2) | **Abusive supervision T1**  (EPRES subscale) | .43^**^ | − | .42^**^ | .31^**^ | .24^**^ | .21^**^ | .22^**^ | .26^**^ | .19^**^ | −.06^*^ | 0.03 | −.15^**^ | −0.05 | .27^**^ | .23^**^ | .28^**^ | .26^**^ | .22^**^ |
| 3) | **Job insecurity T1**  (ERI selected items) | .26^**^ | .39^**^ | − | .15^**^ | .19^**^ | .17^**^ | .15^**^ | .15^**^ | .12^**^ | .04 | .02 | −0.04 | .00 | .21^**^ | .16^**^ | .18^**^ | .15^**^ | .10^**^ |
| 4) | **Job demand T1**  (ERI selected items) | −0.03 | .21^**^ | .18^**^ | − | .10^**^ | .14^**^ | .10^**^ | .13^**^ | .10^**^ | .06^*^ | .06^*^ | −.13^**^ | −.20^**^ | .13^**^ | .12^**^ | .13^**^ | .13^**^ | .07^**^ |
| 5) | **Symptoms of depression T3** (EPDS) | .16^**^ | .24^**^ | .19^**^ | .08^*^ | − | .51^**^ | .60^**^ | .57^**^ | .61^**^ | −.07^*^ | −0.05 | −0.04 | .07^*^ | .53^**^ | .36^**^ | .42^**^ | .38^**^ | .35^**^ |
| 6) | **Symptoms of somatization T3 ^a^** | .10^**^ | .17^**^ | .08^*^ | .12^**^ | .42^**^ | − | .53^**^ | .53^**^ | .46^**^ | −.06^*^ | −0.04 | −.06^*^ | .05 | .36^**^ | .48^**^ | .41^**^ | .36^**^ | .31^**^ |
| 7) | **Symptoms of obsessive-compulsiveness T3 ^a^** | .18^**^ | .25^**^ | .13^**^ | 0.06 | .62^**^ | .52^**^ | − | .63^**^ | .63^**^ | −0.05 | −.06^*^ | −0.01 | .05 | .39^**^ | .34^**^ | .52^**^ | .36^**^ | .32^**^ |
| 8) | **Symptoms of anxiety T3 ^a^** | .11^**^ | .24^**^ | .12^**^ | .08^*^ | .56^**^ | .51^**^ | .68^**^ | − | .51^**^ | −0.04 | −.09^**^ | −0.02 | .06^*^ | .36^**^ | .35^**^ | .40^**^ | .42^**^ | .30^**^ |
| 9) | **Symptoms of anger/hostility T3 ^a^** | .12^**^ | .24^**^ | .08^*^ | .09^*^ | .54^**^ | .44^**^ | .54^**^ | .55^**^ | − | −.06^*^ | −.06^*^ | −0.01 | 0.05 | .37^**^ | .31^**^ | .39^**^ | .32^**^ | .46^**^ |
| 10) | **Age** | −.25^**^ | −0.06 | .09^**^ | .12^**^ | 0.00 | 0.02 | 0.01 | 0.04 | 0.00 | − | .04 | .23^**^ | −.11^**^ | −.07^**^ | −.10^**^ | −.09^**^ | −0.03 | −.11^**^ |
| 11) | **Duration of parental leave up to T3** (months) | 0.05 | 0.05 | 0.00 | −.10^*^ | 0.00 | 0.00 | −0.01 | −0.05 | −0.01 | 0.00 | − | .02 | −.06 | −.04 | −.04 | .01 | −.01 | −.03 |
| 12) | **Academic degree ^b^** | −0.06 | −.13^**^ | −0.02 | −.07^*^ | 0.01 | −.09^*^ | 0.04 | 0.01 | −0.03 | 0.04 | 0.05 | − | −.02 | −.12^**^ | −.12^**^ | −.06^*^ | −.06^*^ | −.05 |
| 13) | **Employment status T1 ^c^** | .34^**^ | −0.04 | 0.05 | −.16^**^ | .12^**^ | 0.05 | .08^*^ | 0.03 | 0.02 | −.18^**^ | 0.05 | 0.05 | − | .05 | .02 | .02 | .02 | .02 |
| 14) | **Symptoms of depression T1**  (EPDS) | .17^**^ | .30^**^ | .28^**^ | .17^**^ | .51^**^ | .27^**^ | .38^**^ | .37^**^ | .28^**^ | −0.03 | −0.01 | −0.02 | 0.03 | − | .41^**^ | .57^**^ | .55^**^ | .53^**^ |
| 15) | **Symptoms of somatization T1 ^a^** | .12^**^ | .23^**^ | .12^**^ | .17^**^ | .29^**^ | .47^**^ | .36^**^ | .34^**^ | .26^**^ | −0.05 | 0.04 | −.11^**^ | 0.04 | .41^**^ | − | .56^**^ | .51^**^ | .43^**^ |
| 16) | **Symptoms of obsessive-compulsiveness T1 ^a^** | .20^**^ | .33^**^ | .23^**^ | .09* | .36^**^ | .29^**^ | .57^**^ | .43^**^ | .29^**^ | −0.03 | 0.01 | 0.00 | .08^*^ | .58^**^ | .51^**^ | − | .64^**^ | .56^**^ |
| 17) | **Symptoms of anxiety T1 ^a^** | .15^**^ | .28^**^ | .19^**^ | .16^**^ | .37^**^ | .33^**^ | .43^**^ | .52^**^ | .32^**^ | −0.01 | 0.00 | 0.01 | 0.06 | .55^**^ | .52^**^ | .63^**^ | − | .50^**^ |
| 18) | **Symptoms of anger/hostility T1 ^a^** | .12^**^ | .30^**^ | .19^**^ | .15^**^ | .29^**^ | .26^**^ | .33^**^ | .36^**^ | .48^**^ | −0.01 | −0.03 | −0.05 | −0.01 | .42^**^ | .32^**^ | .50^**^ | .50^**^ | − |

*Note.* Pearson correlations for mothers (*n* ranges from 1,117 to 1,257 due to missing data) are shown above the diagonal. Pearson correlations for partners (*n* ranges from 641 to 810 due to missing data) are shown below the diagonal. EPRES = Employment Precariousness Scale, ERI = Effort-Reward Imbalance Questionnaire, EPDS = Edinburgh Postnatal Depression Scale.

^a^ Measured with subscales of the Symptom Checklist-90-Revised (SCL-90-R). ^b^ 0 = no academic degree and 1 = academic degree. ^c^ 0 = full-time. 1 = part-time or marginal; if mothers were in employment ban at T1, employment status before employment ban was used.

^*^*p* < .05, ^**^*p* < .01.

**Table S2** Hierarchical multiple regression analyses for mothers with multivariate outliers included

| **Outcomes** | **Predictors** | **Model 1** | | | | | **Model 2** | | | | |
| --- | --- | --- | --- | --- | --- | --- | --- | --- | --- | --- | --- |
| Symptoms of… |  | **B** | **BCa 95% CI** | **β** | ***p*** | ***R*^2^** | **B** | **BCa 95%CI** | **β** | ***p*** | ***R*^2^** |
| **Depression** | Precarious employment**^a^** | 1.79 | [1.18,2.42] | .19 | **<.001** | .04 | 0.71 | [0.18,1.25] | .08 | ***.008*** | .28 |
|  | Abusive supervision | 1.00 | [0.61,1.42] | .17 | **<.001** | .08 | 0.49 | [0.16,0.83] | .08 | **.004** |  |
|  | Job insecurity | 0.38 | [0.18,0.58] | .13 | **<.001** |  | 0.20 | [0.02,0.37] | .07 | ***.024*** |  |
|  | Job demand | 0.12 | [−0.05,0.30] | .04 | .178 |  | 0.02 | [−014,0.17] | .01 | .848 | .30 |
| **Somatization** | Precarious employment**^a^** | 1.65 | [1.05,2.31] | .19 | **<.001** | .04 | 1.00 | [0.50,1.52] | .12 | **<.001** | .24 |
|  | Abusive supervision | 0.64 | [0.29,1.01] | .13 | **<.001** |  | 0.25 | [−0.05,0.55] | .05 | *.117* |  |
|  | Job insecurity | 0.30 | [0.12,0.49] | .11 | **.004** |  | 0.19 | [0.03,0.35] | .07 | ***.026*** |  |
|  | Job demand | 0.26 | [0.09,0.44] | .10 | **.002** | .06 | 0.19 | [0.04,0.34] | .07 | **.013** | .24 |
| **Obsessive-compulsiveness** | Precarious employment**^a^** | 2.16 | [1.46,3.00] | .22 | **<.001** | .05 | 1.06 | [0.48,1.65] | .11 | **<.001** | .30 |
|  | Abusive supervision | 1.02 | [0.60,1.47] | .17 | **<.001** |  | 0.37 | [−0.01,0.79] | .06 | *.066* |  |
|  | Job insecurity | 0.28 | [0.06,0.50] | .09 | .**013** |  | 0.15 | [−0.04,0.35] | .05 | .128 |  |
|  | Job demand | 0.13 | [−0.04,0.30] | .04 | .141 | .06 | 0.04 | [−0.11,0.20] | .01 | .584 | .28 |
| **Anxiety** | Precarious employment**^a^** | 1.27 | [0.78,1.79] | .20 | **<.001** | .05 | 0.82 | [0.38,0.31] | .13 | **<.001** | .22 |
|  | Abusive supervision | 0.69 | [0.42,1.00] | .19 | **<.001** |  | 0.36 | [0.12,0.63] | .10 | **.005** |  |
|  | Job insecurity | 0.15 | [0.02,0.28] | .08 | *.****033*** |  | 0.11 | [−0.01,0.23] | .06 | .093 |  |
|  | Job demand | 0.13 | [0.03,0.24] | .07 | **.018** | .07 | 0.07 | [−0.02,0.17] | .04 | .127 | .22 |
| **Anger/hostility** | Precarious employment**^a^** | 1.12 | [0.71,1.55] | .19 | **<.001** | .04 | 0.71 | [0.37,1.07] | .12 | **<.001** | .21 |
|  | Abusive supervision | 0.58 | [0.32,0.86] | .16 | **<.001** |  | 0.27 | [0.04,0.52] | .07 | **.033** |  |
|  | Job insecurity | 0.10 | [−0.03,0.23] | .05 | .142 |  | 0.08 | [−0.04,0.21] | .05 | .178 |  |
|  | Job demand | 0.11 | [0.01,0.21] | .06 | ***.042*** | .05 | 0.08 | [−0.01,0.18] | .05 | .090 | .23 |

*Note*. Results of multiple regression analyses with working conditions and sociodemographic confounders age, duration of parental leave, academic degree, and employment status (Model 1) and pre-existing (T1) symptoms of respective mental health outcomes added in the second step (Model 2). *R*^2^ = adjusted. Working conditions were measured during pregnancy (T1) and mental health outcomes 14 months after childbirth (T3). For analyses including confounding variables see Additional file 4, Tables 4–8. BCa 95% CI = Bias-corrected and accelerated 95% bootstrap confidence interval.

^a^ Precarious employment was analyzed in a separate regression model.

*p*-values and BCa CI based on 5,000 bootstrap samples. Significant *p*-values (*p* < .05) are presented in bold. Significances deviating from analyses with multivariate outliers excluded are presented in italics.

**Table S3** Hierarchical multiple regression analyses for partners with multivariate outliers included

| **Outcomes** | **Predictors** | **Model 1** | | | | | **Model 2** | | | | |
| --- | --- | --- | --- | --- | --- | --- | --- | --- | --- | --- | --- |
| Symptoms of… |  | **B** | **BCa 95% CI** | **β** | ***p*** | ***R*^2^** | **B** | **BCa 95%CI** | **β** | ***p*** | ***R*^2^** |
| **Depression** | Precarious employment**^a^** | 1.17 | [0.49,1.88] | .14 | **<.001** | .02 | 0.45 | [−0.15,1.07] | .05 | .148 | .24 |
|  | Abusive supervision | 1.25 | [0.72,1.82] | .21 | **<.001** | .09 | 0.63 | [0.14,1.14] | .10 | **.008** |  |
|  | Job insecurity | 0.29 | [0.07,0.50] | .10 | **.009** |  | 0.06 | [−0.14,0.26] | .02 | .582 |  |
|  | Job demand | 0.11 | [−0.09,0.29] | .04 | .298 |  | −0.01 | [−0.18,0.16] | .00 | .899 | .27 |
| **Somatization** | Precarious employment**^a^** | 0.71 | [0.12,1.30] | .10 | **.018** | .01 | 0.34 | [−0.23,0.95] | .05 | .240 | .21 |
|  | Abusive supervision | 0.79 | [0.38,1.24] | .16 | **<.001** |  | 0.35 | [0.00,0.72] | .07 | *.058* |  |
|  | Job insecurity | 0.00 | [−0.18,0.18] | .00 | .994 |  | −0.02 | [−0.18,0.15] | −.01 | .834 |  |
|  | Job demand | 0.23 | [0.05,0.41] | .10 | ***.017*** | .04 | 0.10 | [−0.05,0.25] | .04 | .209 | .23 |
| **Obsessive-compulsiveness** | Precarious employment**^a^** | 1.69 | [0.98,2.38] | .18 | **<.001** | .03 | 0.74 | [0.13,1.34] | .08 | .**018** | .30 |
|  | Abusive supervision | 1.67 | [1.20,2.20] | .26 | **<.001** |  | 0.62 | [0.15,1.12] | .10 | **.010** |  |
|  | Job insecurity | 0.08 | [−0.12,0.29] | .03 | .448 |  | −0.10 | [−0.29,0.09] | −.03 | .284 |  |
|  | Job demand | 0.06 | [−0.14,0.26] | .02 | .574 | .08 | 0.04 | [−0.12,0.21] | .01 | .644 | .34 |
| **Anxiety** | Precarious employment**^a^** | 0.62 | [0.27,1.00] | .12 | **<.001** | .01 | 0.27 | [−0.04,0.58] | .05 | .106 | .24 |
|  | Abusive supervision | 0.87 | [0.57,1.21] | .24 | **<.001** |  | 0.44 | [0.15,0.74] | .12 | **.004** |  |
|  | Job insecurity | 0.05 | [−0.07,0.16] | .03 | .442 |  | −0.02 | [−0.13,0.10] | −.01 | .780 |  |
|  | Job demand | 0.06 | [−0.06,0.18] | .04 | .342 | .06 | −0.02 | [−0.13,0.09] | −.01 | .673 | .28 |
| **Anger/hostility** | Precarious employment**^a^** | 0.67 | [0.25,1.11] | .13 | **.005** | .01 | 0.31 | [−0.07,0.68] | .06 | .105 | .23 |
|  | Abusive supervision | 0.85 | [0.51,1.23] | .23 | **<.001** |  | 0.41 | [0.11,0.74] | .11 | **.019** |  |
|  | Job insecurity | −0.04 | [−0.17,0.09] | −.02 | .525 |  | −0.09 | [−0.21,0.03] | −.05 | .142 |  |
|  | Job demand | 0.06 | [−0.06,0.18] | .04 | .292 | .05 | 0.00 | [−0.10,0.10] | .00 | .989 | .23 |

*Note*. Results of multiple regression analyses with working conditions and sociodemographic confounders academic degree and employment status (Model 1) and pre-existing (T1) symptoms of respective mental health outcomes added in the second step (Model 2). *R*^2^ = adjusted. Working conditions were measured during pregnancy (T1) and mental health outcomes 14 months after childbirth (T3). For analyses including confounding variables see Additional file 4, Tables 4–8. BCa 95% CI = Bias-corrected and accelerated 95% bootstrap confidence interval.

^a^ Precarious employment was analyzed in a separate regression model.

*p*-values and BCa CI based on 5,000 bootstrap samples. Significant *p*-values (*p* < .05) are presented in bold. Significances deviating from analyses with multivariate outliers excluded are presented in italics.

## Summary of deviations from analyses with outliers excluded

**Precarious employment**: The conducted sensitivity analyses with outliers included (see Tables 2 and 3) revealed some significant differences in Model 2: Precarious employment remained a significant predictor for symptoms of depression in mothers (β = .08, *p* = .008) but no longer significantly predicted symptoms of anxiety in partners (β = .05, *p* = .106).

**Abusive supervision:** The conducted sensitivity analyses with outliers included (see Tables 2 and 3) revealed some significant differences in Model 2: Abusive supervision no longer significantly predicted symptoms of somatization (β = .05, *p* = .117) and obsessive-compulsiveness (β = .06, *p* = .066) in mothers and symptoms of somatization in partners (β = .07, *p* = .058).

**Job insecurity:** The sensitivity analyses with outliers included (see Tables 2 and 3) yielded similar results for partners. In mothers, job insecurity became a significant predictor for symptoms of anxiety (β = .08, *p* = .033) in Model 1 and remained a significant predictor for symptoms of depression (β = .07, *p* = .024) and somatization (β = .07, *p* = .026) in Model 2.

**Job demand:** The conducted sensitivity analyses with outliers included (see Tables 2 and 3) revealed some significant differences in Model 1: Job demand became a significant predictor for symptoms of anger/hostility in mothers (β = .06, *p* = .042), and symptoms of somatization in partners (β = .10, *p* = .017).

**Table S4** Hierarchical multiple regression analyses predicting T3 symptoms of depression from T1 working conditions and baseline symptoms of depression (outliers included)

|  | Mothers | | | | Partners | | | |
| --- | --- | --- | --- | --- | --- | --- | --- | --- |
|  | Model 1a | Model 2a | Model 1b | Model 2b | Model 1a | Model 2a | Model 1b | Model 2b |
| Age | −.01 | −.02 | −.04^*^ | −.03 | x | x | x | x |
| Duration of parental leave up to T3 | −.03 | −.02 | −.06 | −.03 | x | x | x | x |
| Academic degree ^a^ | .01 | .06^*^ | .01 | *.05^*^* | .01 | .01 | .04 | .03 |
| Employment status T1 ^b^ | .04 | .03 | *.08^**^* | .05 | .06 | .08 | .13^**^ | .11^**^ |
| Symptoms of depression T1 (EPDS) | x | .51^**^ | x | .49^**^ | x | .47^**^ | x | .47^**^ |
| Precarious employment (EPRES) | .19^**^ | *.07^**^* | x | x | .14^**^ | .05 | x | x |
| Abusive supervision (EPRES subscale) | x | x | .17^**^ | .08^**^ | x | x | .21^**^ | .10^**^ |
| Job insecurity (ERI subscale) | x | x | .13^**^ | *.07^*^* | x | x | .10^**^ | .02 |
| Job demand (ERI subscale) | x | x | .04 | .01 | x | x | .04 | .00 |
| *R*² (adjusted) | .04 | .29 | .08 | .30 | .02 | .24 | .09 | .27 |

*Note*. Standardized βs are reported. Hierarchical multiple regression analyses with working condition(s) and sociodemographic confounders age (only mothers), duration of parental leave (only mothers), educational level, and employment status entered in a first step (Model 1) and baseline (T1) symptoms of respective mental health outcomes added in the second step (Model 2). In Model 1a and 2a, precarious employment was entered as working condition; in Model 1b and 2b, abusive supervision, job insecurity, and job demand were entered simultaneously as working conditions. Symptoms of depression were measured with the Edinburgh Postnatal Depression Scale (EPDS). T1 = during pregnancy, T3 = 14 months postpartum. EPRES = Employment Precariousness Scale, ERI = Effort-Reward Imbalance Questionnaire.

^a^ 0 = no academic degree and 1 = academic degree. ^b^ 0 = full-time, 1 = part-time or marginal; if mothers were in employment ban at T1, employment status/working hours before employment ban was used.

^*^*p* < .05; ^**^*p* < .01; *p*-values based on 5,000 bootstrap samples. Significances deviating from analyses with multivariate outliers excluded are presented in italics.

**Table S5** Hierarchical multiple regression analyses predicting T3 symptoms of somatization from T1 working conditions and baseline symptoms of somatization (outliers included)

|  | Mothers | | | | Partners | | | |
| --- | --- | --- | --- | --- | --- | --- | --- | --- |
|  | Model 1a | Model 2a | Model 1b | Model 2b | Model 1a | Model 2a | Model 1b | Model 2b |
| Age | −.01 | .02 | −.05 | −.01 | x | x | x | x |
| Duration of parental leave up to T3 | −.03 | −.01 | −.04 | −.02 | x | x | x | x |
| Academic degree ^a^ | −.02 | .01 | −.02 | .01 | −.07 | −.03 | −.06 | −.03 |
| Employment status T1 ^b^ | .02 | .03 | .06 | .05 | .01 | .01 | *.07^*^* | .04 |
| Symptoms of somatization T1 (SCL-90-R) | x | .46^**^ | x | .44^**^ | x | .45^**^ | x | .45^**^ |
| Precarious employment (EPRES) | .19^**^ | .12^**^ | x | x | .10^*^ | .05 | x | x |
| Abusive supervision (EPRES subscale) | x | x | .13^**^ | *.05* | x | x | .16^**^ | *.07* |
| Job insecurity (ERI subscale) | x | x | .11^**^ | *.07^*^* | x | x | .00 | −.01 |
| Job demand (ERI subscale) | x | x | .10^**^ | .07^*^ | x | x | *.10^*^* | .04 |
| *R*² (adjusted) | .04 | .24 | .06 | .24 | .01 | .21 | .04 | .23 |

*Note*. Standardized βs are reported. Hierarchical multiple regression analyses with working condition(s) and sociodemographic confounders age (only mothers), duration of parental leave (only mothers), educational level, and employment status entered in a first step (Model 1) and baseline (T1) symptoms of respective mental health outcomes added in the second step (Model 2). In Model 1a and 2a, precarious employment was entered as working condition; in Model 1b and 2b, abusive supervision, job insecurity, and job demand were entered simultaneously as working conditions. Symptoms of somatization were measured with the corresponding subscale of the Symptom Checklist-90-Revised (SCL-90-R). T1 = during pregnancy, T3 = 14 months postpartum. EPRES = Employment Precariousness Scale, ERI = Effort-Reward Imbalance Questionnaire.

^a^ 0 = no academic degree and 1 = academic degree. ^b^ 0 = full-time, 1 = part-time or marginal; if mothers were in employment ban at T1, employment status/working hours before employment ban was used.

^*^*p* < .05; ^**^*p* < .01; *p*-values based on 5,000 bootstrap samples. Significances deviating from analyses with multivariate outliers excluded are presented in italics.

**Table S6** Hierarchical multiple regression analyses predicting T3 symptoms of obsessive-compulsiveness (OC) from T1 working conditions and baseline symptoms of obsessive-compulsiveness (outliers included)

|  | Mothers | | | | Partners | | | |
| --- | --- | --- | --- | --- | --- | --- | --- | --- |
|  | Model 1a | Model 2a | Model 1b | Model 2b | Model 1a | Model 2a | Model 1b | Model 2b |
| Age | −.01 | .01 | −.04 | .00 | x | x | x | x |
| Duration of parental leave up to T3 | −.04 | −.05 | −.06 | *−.06^*^* | x | x | x | x |
| Academic degree ^a^ | .04 | .05 | .03 | .03 | .06 | .05 | .08^*^ | .05 |
| Employment status T1 ^b^ | .01 | .02 | .06^*^ | .05 | .00 | −.01 | *.09^*^* | .05 |
| Symptoms of OC T1 (SCL-90-R) | x | .51^**^ | x | .50^**^ | x | .52^**^ | x | .54^**^ |
| Precarious employment (EPRES) | .22^**^ | .11^**^ | x | x | .18^**^ | .08^*^ | x | x |
| Abusive supervision (EPRES subscale) | x | x | .17^**^ | .06 | x | x | .26^**^ | .10^**^ |
| Job insecurity (ERI subscale) | x | x | .09^*^ | .05 | x | x | .03 | −.03 |
| Job demand (ERI subscale) | x | x | .04 | .01 | x | x | .02 | .01 |
| *R*² (adjusted) | .05 | .30 | .06 | .28 | .03 | .30 | .08 | .34 |

*Note*. Standardized βs are reported. Hierarchical multiple regression analyses with working condition(s) and sociodemographic confounders age (only mothers), duration of parental leave (only mothers), educational level, and employment status entered in a first step (Model 1) and baseline (T1) symptoms of respective mental health outcomes added in the second step (Model 2). In Model 1a and 2a, precarious employment was entered as working condition; in Model 1b and 2b, abusive supervision, job insecurity, and job demand were entered simultaneously as working conditions. Symptoms of OC were measured with the corresponding subscale of the Symptom Checklist-90-Revised (SCL-90-R). T1 = during pregnancy, T3 = 14 months postpartum. EPRES = Employment Precariousness Scale, ERI = Effort-Reward Imbalance Questionnaire.

^a^ 0 = no academic degree and 1 = academic degree. ^b^ 0 = full-time, 1 = part-time or marginal; if mothers were in employment ban at T1, employment status/working hours before employment ban was used.

^*^*p* < .05; ^**^*p* < .01; *p*-values based on 5,000 bootstrap samples. Significances deviating from analyses with multivariate outliers excluded are presented in italics.

**Table S7** Hierarchical multiple regression analyses predicting T3 symptoms of anxiety from T1 working conditions and baseline symptoms of anxiety (outliers included)

|  | Mothers | | | | Partners | | | |
| --- | --- | --- | --- | --- | --- | --- | --- | --- |
|  | Model 1a | Model 2a | Model 1b | Model 2b | Model 1a | Model 2a | Model 1b | Model 2b |
| Age | .01 | .00 | −.02 | −.01 | x | x | x | x |
| Duration of parental leave up to T3 | −.08 | −.08^*^ | *−.10^*^* | −.09^*^ | x | x | x | x |
| Academic degree ^a^ | .02 | .04 | .02 | .03 | .04 | .03 | .04 | .02 |
| Employment status T1 ^b^ | .03 | .04 | .09^**^ | .08^*^ | −.02 | −.03 | .04 | .00 |
| Symptoms of anxiety T1 (SCL-90-R) | x | .42^**^ | x | .40^**^ | x | .48^**^ | x | .49^**^ |
| Precarious employment (EPRES) | .20^**^ | .13^**^ | x | x | .12^**^ | *.05* | x | x |
| Abusive supervision (EPRES subscale) | x | x | .19^**^ | .10^**^ | x | x | .24^**^ | .12^**^ |
| Job insecurity (ERI subscale) | x | x | *.08^*^* | .06 | x | x | .03 | −.01 |
| Job demand (ERI subscale) | x | x | .07^*^ | .04 | x | x | .04 | −.01 |
| *R*² (adjusted) | .05 | .22 | .07 | .22 | .01 | .24 | .06 | .28 |

*Note*. Standardized βs are reported. Hierarchical multiple regression analyses with working condition(s) and sociodemographic confounders age (only mothers), duration of parental leave (only mothers), educational level, and employment status entered in a first step (Model 1) and baseline (T1) symptoms of respective mental health outcomes added in the second step (Model 2). In Model 1a and 2a, precarious employment was entered as working condition; in Model 1b and 2b, abusive supervision, job insecurity, and job demand were entered simultaneously as working conditions. Symptoms of anxiety were measured with the corresponding subscale of the Symptom Checklist-90-Revised (SCL-90-R). T1 = during pregnancy, T3 = 14 months postpartum. EPRES = Employment Precariousness Scale, ERI = Effort-Reward Imbalance Questionnaire.

^a^ 0 = no academic degree and 1 = academic degree. ^b^ 0 = full-time, 1 = part-time or marginal; if mothers were in employment ban at T1, employment status/working hours before employment ban was used.

^*^*p* < .05; ^**^*p* < .01; *p*-values based on 5,000 bootstrap samples. Significances deviating from analyses with multivariate outliers excluded are presented in italics.

**Table S8** Hierarchical multiple regression analyses predicting T3 symptoms of anger/hostility from T1 working conditions and baseline symptoms of anger/hostility (outliers included)

|  | Mothers | | | | Partners | | | |
| --- | --- | --- | --- | --- | --- | --- | --- | --- |
|  | Model 1a | Model 2a | Model 1b | Model 2b | Model 1a | Model 2a | Model 1b | Model 2b |
| Age | −.02 | .00 | *−.05* | −.01 | x | x | x | x |
| Duration of parental leave up to T3 | −.06 | −.05 | *−.08^*^* | −.06 | x | x | x | x |
| Academic degree ^a^ | .02 | .03 | .03 | .03 | −.01 | .01 | .01 | .01 |
| Employment status T1 ^b^ | .02 | .03 | .06 | .05 | −.04 | −.02 | .04 | .03 |
| Symptoms of anger/hostility T1 (SCL-90-R) | x | .42^**^ | x | .43^**^ | x | .48^**^ | x | .45^**^ |
| Precarious employment (EPRES) | .19^**^ | .12^**^ | x | x | .12^**^ | .06 | x | x |
| Abusive supervision (EPRES subscale) | x | x | .16^**^ | .07^*^ | x | x | .23^**^ | .11^*^ |
| Job insecurity (ERI subscale) | x | x | .05 | .05 | x | x | −.02 | −.05 |
| Job demand (ERI subscale) | x | x | *.06^*^* | .05 | x | x | .04 | .00 |
| *R*² (adjusted) | .04 | .21 | .05 | .23 | .01 | .23 | .05 | .23 |

*Note*. Standardized βs are reported. Hierarchical multiple regression analyses with working condition(s) and sociodemographic confounders age (only mothers), duration of parental leave (only mothers), educational level, and employment status entered in a first step (Model 1) and baseline (T1) symptoms of respective mental health outcomes added in the second step (Model 2). In Model 1a and 2a, precarious employment was entered as working condition; in Model 1b and 2b, abusive supervision, job insecurity, and job demand were entered simultaneously as working conditions. Symptoms of anger/hostility were measured with the corresponding subscale of the Symptom Checklist-90-Revised (SCL-90-R). T1 = during pregnancy, T3 = 14 months postpartum. EPRES = Employment Precariousness Scale, ERI = Effort-Reward Imbalance Questionnaire.

^a^ 0 = no academic degree and 1 = academic degree. ^b^ 0 = full-time, 1 = part-time or marginal; if mothers were in employment ban at T1, employment status/working hours before employment ban was used.

^*^*p* < .05; ^**^*p* < .01; *p*-values based on 5,000 bootstrap samples. Significances deviating from analyses with multivariate outliers excluded are presented in italics.
